# Folding intermediate states of the parallel human telomeric G-quadruplex DNA explored using Well-Tempered Metadynamics

**DOI:** 10.1038/s41598-020-59774-x

**Published:** 2020-02-21

**Authors:** Roberta Rocca, Ferruccio Palazzesi, Jussara Amato, Giosuè Costa, Francesco Ortuso, Bruno Pagano, Antonio Randazzo, Ettore Novellino, Stefano Alcaro, Federica Moraca, Anna Artese

**Affiliations:** 1 Dipartimento di Scienze della Salute, Università “Magna Græcia” di Catanzaro, Campus Salvatore Venuta, Viale Europa, 88100 Catanzaro, Italy; 2 Net4Science srl, Università “Magna Græcia” di Catanzaro, Campus Salvatore Venuta, Viale Europa, 88100 Catanzaro, Italy; 3 Research Informatics, Computational Chemistry & Cheminformatics, Aptuit an Evotec Company, Via A. Fleming 4, 37135 Verona, Italy; 4 Department of Pharmacy, University of Naples “Federico II”, Via D. Montesano 49, Naples, 80131 Italy

**Keywords:** Computational chemistry, Drug development

## Abstract

An increasingly comprehension of the folding intermediate states of DNA G-quadruplexes (G4s) is currently an important scientific challenge, especially for the human telomeric (*h-te*l) G4s-forming sequences, characterized by a highly polymorphic nature. Despite the G-triplex conformation was proposed as one of the possible folding intermediates for the antiparallel and hybrid *h-tel* G4s, for the parallel *h-tel* topology with an all-*anti* guanine orientation, a vertical strand-slippage involving the G-triplets was proposed in previous works through microseconds-long standard molecular dynamics simulations (MDs). Here, in order to get further insights into the vertical strand-slippage and the folding intermediate states of the parallel *h-tel* G4s, we have carried out a Well-Tempered Metadynamics simulation (WT-MetaD), which allowed us to retrieve an ensemble of six G4s having two/G-tetrad conformations derived by the G-triplets vertical slippage. The insights highlighted in this work are aimed at rationalizing the mechanistic characterisation of the parallel *h-tel* G4 folding process.

## Introduction

**Figure 1:**
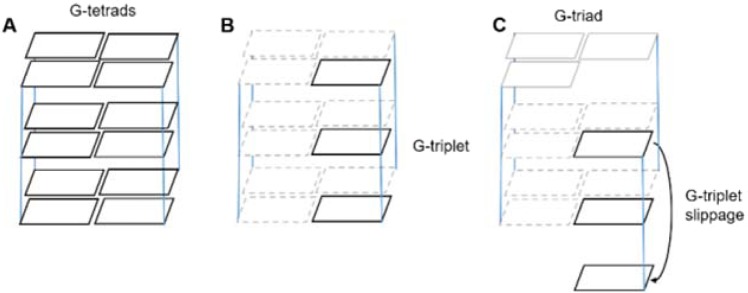
Schematic representation of: (**A**) the three planes of the G-tetrad core typical of G4s; (**B**) the G-triplet; (**C**) the vertical slippage movement involving the G-triplet, which leads the formation of G4s having one G-triad and two/G-tetrad planes. For clarity, the G-triad residues are depicted with solid gray lines, while the guanine residues of the two/G-tetrad planes that are not involved in the G-triplet slippage are shown as dashed gray lines.

G-quadruplexes^1,2^ (G4s) are non canonical DNA structures present at the telomere ends and involved in the genome stability and in the inhibition of the telomerase enzyme, a reverse transcriptase responsible for the cellular immortalization^3,4^ and up-regulated in ~85% of human tumors^3^. The evidence that G4s formation at the 3′-end of telomere DNA can effectively hamper telomerase from adding further repeats^3^ suggests G4s as a promising target for anticancer therapy^5–7^. In order to identify new compounds as potential anticancer drugs, the structure-based drug design targeting G4s could be a crucial step in gaining accurate information about the structural features of G4s^8–13^. Unfortunately, the rational design of novel G4 binders is hampered both by the structural polymorphism of these non-canonical conformations and by the lack of a well-defined ligand binding site. Therefore, enhanced sampling methods, like Metadynamics, have been required in order to deeply define the ligand binding mode^14,15^. Monomolecular G4s of the human telomeric sequence *d*[AG_3_(TTAGGG)_3_] (*h-tel*) can adopt different folding topologies according to the relative orientations of the four strands^16^. In fact, while the arrangement of the *h-tel* monomolecular G4s has been well characterized by NMR in Na^+^ to adopt a basket-type conformation with the single strands assuming an antiparallel orientation^17^, in presence of K^+^ ions the folding topology of the *h-tel* monomolecular G4s includes both parallel-stranded and hybrid (mixed parallel/antiparallel) conformations, raising the question of which conformation is biologically more favoured. In 2006, the NMR hybrid *syn/anti* conformation was proposed as the most favoured one under K^+^ physiological conditions^18^, whose folding organization is different from that reported in K^+^ ions by crystallography consisting, instead, in the parallel-stranded topology with three G-tetrads and three symmetrical external *d*(TTA) loops in a propeller-like arrangement^19^, considered not predominant in solution^20^. Later, in 2007 Xue *et al*. reported that the same parallel-stranded conformation found by X-ray is adopted also in K^+^ solution under molecular crowding conditions^21^. Given the molecular crowding nature of the intracellular environment^22^, these observations led to the hypothesis that the propeller-type parallel-stranded conformation might be more biologically favoured under such conditions. These evidences were confirmed in 2011 with the first NMR *h-tel* G4s in K^+^ containing crowded solutions, partially keeping a structural similarity with the crystallographic conformation^23^. Computational and experimental studies about the kinetics and folding pathways have suggested the formation of a G-triplex structure with three G-triad planes, as plausible intermediate state in the G4 folding pathway of the antiparallel^24,25^ and both hybrid-1 and hybrid-2 topologies^26–29^. Fewer efforts have been dedicated, instead, to define the intermediate states of the propeller-type parallel topology of the native human telomere sequence for which the G-triplex intermediate has been not experimentally identified yet. The most recent example for the parallel propeller-type folding was probed through microsecond-scale molecular dynamics simulations (MDs) performed on a truncated sequence (*d*[A(G_3_T_2_A)_2_G_3_]) of the parallel propeller-like G4. On the basis of such simulation data, it was suggested that G-triplexes with all*-anti* guanines conformation are quite unstable and therefore unlikely to contribute to the folding of the human telomere G4, but rather to its unfolding. For the all-*anti* guanine monomolecular (and tetramolecular) G4s (Fig. 1A), the most natural arrangement is the vertical strand-slippage movement involving the G-triplets (Fig. 1B,C), that leads to a reduced number of G-tetrad planes (two G-tetrads and one G-triad), as demonstrated by standard MDs. By contrast, the same arrangement is prevented by the steric conflict between the alternation of *syn-* and *anti-* nucleotides in the hybrid and antiparallel topologies^30,31^.

All the above reported examples suggest the existence of other possible intermediate substrates perhaps with specific cellular functions. However, despite the large amount of all-atom computer simulations performed so far, a detailed atomistic picture of the folding process of the parallel monomolecular G4s is still unclear due to the time scale limitations of unrestrained standard simulations. To this aim, the use of enhanced sampling methods is mandatory.

In the light of the previous findings^30,31^, we have investigated the folding process of the native 22-nt monomolecular DNA G4 *d*[AGGG(TTAGGG)_3_] (PDB ID: 1KF1)^19^, considered as the most probable at cellular level^21,32^, through Well-Tempered Metadynamics simulations (WT-MetaD) in explicit solvent and K^+^ ions. In this way, the time-scale limitation problem is overcome and the so-called “rare events” are accelerated^33^. The aim of our work was to obtain a clear scenery of the possible vertical slippage combinations and the identification of the most relevant ensemble structures deriving from each arrangement. It is worth to underline that, in K^+^ solutions, NMR data indicate *d*[AGGG(TTAGGG)_3_] sequence as an equilibrium mixture of more than one conformer^34^, making its experimental determination impossible under usual conditions. Nevertheless, an experimental structure of a G-rich sequence, having one G-triad and two G-tetrad planes and derived by the G-triplet slippage, has been recently retrieved by NMR (PDB ID: 2N60)^35^. Based on these observations, here we analysed the folding process of the G4 parallel topology, by including further insights of the vertical slippage mechanism and of the formation of all the possible ensemble conformations. The results presented in this work could help to integrate the current knowledge on the G4 folding processes, which are essential to stabilize the nucleic structures and to understand their formation in the genome.

## Results

### The free energy surface (FES) of the parallel DNA G4

**Figure 2:**
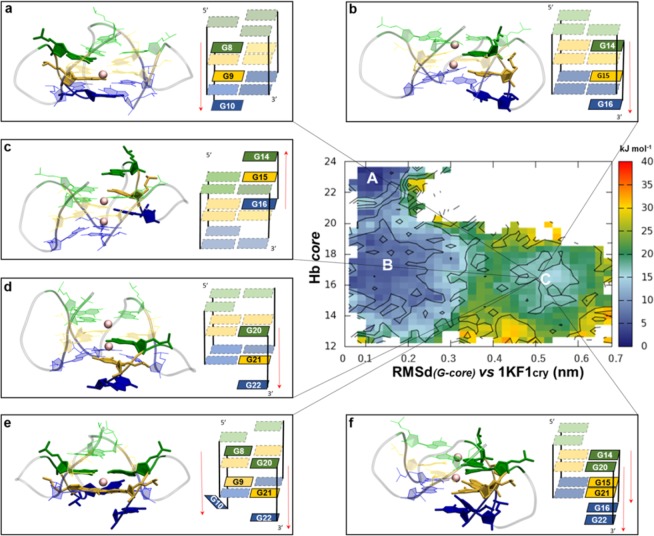
3D structures and schematic representations of the slipped ensemble structures found in the basin C from the reweighted FES. (**a**) **2d** conformation derived from the vertical slippage of the second G-triplet (G8:G9:G10) towards the 3′-end position. (**b**) **3d** conformation characterized by the slippage of the third G-triplet (G14:G15:G16) towards the 3′-end position. (**c**) **3u** structure formed by the slippage of the third G-triplet (G14:G15:G16) towards the 5′-end position. (**d**) **4d** conformation in which the fourth G-triplet (G20:G21:G22) moves towards the 3′-end position. (**e,f**) **24d** and **34d** conformations derived from the simultaneous slippage of the fourth G-triplet (G20:G21:G22) with the second (G8:G9:G10) and the third (G14:G15:G16) G-triplets, respectively. For clarity, the G-triplets not involved in the vertical slippage are shown in transparency with dashed gray lines. The red arrows point out the direction of the movement.

The crystal structure of the *h-tel* (PDB ID: 1KF1)^19^ was considered for this study. Two different CVs were biased: the first CV (π-π_core_), introduced by Giberti *et al*. to study the nucleation of urea crystals^36^, here was employed to describe the π-π stacking among the guanine residues of the G-tetrads (for details, see Supplementary Information, including Figure S1); the second was a coordination CV (Hb_core_ 1KF1), suitable to count the total number of the native Hoogsteen hydrogen-bonds network holding the three G-tetrad planes (for details, see the Supplementary Information, including Tables S1 and S2). The vertical slippage of the G-triplets was observed during 140 ns of WT-MetaD simulation. The recrossing events occurring with the selected biased CVs (Supplementary Information, Figure S2A,B), indicate those CVs as good descriptors of the reaction, since they were able to distinguish the three different energetic states corresponding to the three different minima A, B and C resulting from the Free Energy Surface (FES) (Supplementary Information, Figure S3). Unfortunately, the different conformations found in the two minima B and C were not completely distinguishable in the CVs space, since a partial overlap of the populations was observed as shown in the plots of Figure S3i and Figure S3h, respectively. Since this could lead not only to the lack of the convergence, but also to an inaccuracy in the calculation of the Free Energy value of each slipped structure, we reweighted the FES as function of two unbiased CVs (discussed in the Methods section). This was done by means of the reweighting algorithm that allows calculating the unbiased probabilities from the biased probabilities along the CV and finally reconstructing the FES from the unbiased probabilities^37^. Looking at the reweighted FES reported in Fig. 2, three main energetic minima were identified. The basin A is the energetically deepest one and it corresponds to parallel G4 motifs with a folding arrangement very similar to that of the 1KF1_*Cry*_. The second minimum, basin B, is populated by transition-state structures (TS-structures) with a slightly more unstable barely stacked conformations of G4s characterized by a partial distancing of the guanine residues of the G-tetrad planes. Finally, the most interesting basin C retrieves an ensemble of two G-tetrad planes structures arising from the vertical slippage movement of different G-triplets. Specifically, on the basis of which G-triplet is involved in the vertical slippage and the movement direction, globally six ensembles were identified. A detailed description of basins A and B is addressed in the Supplementary Information (Figures S4–S6 and Table S3), while results retrieved from basin C are reported in the following paragraphs. The convergence of the WT-MetaD simulation is shown in Figure S7 and S8 of the Supplementary Information.

### The slipped G4s structures are observed in basin C

At variance with basins A and B, basin C was the energetically highest. It depicted an ensemble of six G4 arrangements with only two G-tetrad planes coming from the vertical slippage movement of different guanine triplets (G-triplets) (Table 1). For clarity reasons, we structurally differentiated each vertical slippage arrangement with an alphanumeric code. Specifically, numbers correspond to the G-triplets involved in the vertical movement, following the 5′-to-3′ direction, while letters explain the direction of the vertical slippage: “*u*” (*up*) and “*d*” (*down*), when the G-triplet slips towards the 5′-end and the 3′-end positions, respectively (Table 1). Moreover, as reported in Table S2, such structures were also extracted from the basin C through a plumed tool relying on the number of the native Hbcore engaged within the two G-tetrad planes. Their 3D schematic representation is shown in Fig. 2, while their percentage of existence relative to basin C was reported as histograms in the Supplementary Information (Figure S9).

**Table 1:** G-triplets residues involved in the vertical slippage for each structure found in the basin C. The guanine residues forming the two G-tetrad planes and the G-triads are also reported.

Basin C structures	Slipped G-Triplet Residues	Two G-tetrads residues	G-triad residues
**2d**	5′-G8:G9:G10-3′ *5*′*→3*′	G3:**G8**:G15:G21 G4:**G9**:G16:G22	G2:G14:G20
**3d**	5′-G14:G15:G16-3′ *5*′*→3*′	G3:G9:**G14**:G21 G4:G10:**G15**:G22	G2:G8:G20
**3u**	3′-G16:G15:G14-5′ *3*′*→5*′	G2:G8:**G15**:G20 G3:G9:**G16**:G21	G4:G10:G22
**4d**	5′-G20:G21:G22-3′ *5*′*→3*′	G3:G9:G15:**G20** G4:G10:G16:**G21**	G2:G8:G14
**24d**	5′-G8:G9:G10-3′ 5′-G20:G21:G22-3′ 5′→3′	G3:**G8**:G14:**G20** G4:**G9**:G15:**G21**	No G-triad
**34d**	5′-G14:G15:G16-3′ 5′-G20:G21:G22-3′ *5*′*→3*′	G3:G9:**G14**:**G20** G4:G10:**G15**:G21	No G-triad

As it can be observed from Fig. 2, most of these structures were characterized by the slippage of a single strand of guanines, in which the G-triplets (in the **2d**, **3d** and **4d** ensembles) moved towards the 3′-end position, thus leading to the disruption of the expected Hoogsteen hydrogen bonds network (Hb_core_), as typically found in the G-tetrad of 1KF1_*cry*_ (Movie S1). This event led to the formation of a G-triad at the 5′-end (Fig. 2a,b,d) with a vacant site of the slipped guanine highly accessible to the water solvent. It is worth to mention that a similar G-triad conformation was previously retrieved with NMR by Heddi and co-workers^35^. Subsequently, Stefl *et al*. described the same structure for the tetramolecular *d*(TGGGGT)_4_ G4, in which the G-triad arrange in an “*open*” and “*closed*” form^38^. In our study, we also disclosed the formation of both the G-triad forms in the parallel monomolecular *h-tel* G4 (see Supplementary Information Figure S10A,B). In particular, the “open” G-triad was detected in the **3d**, **3u** and **4d** conformations and it was characterized by the conservation of the Hoogsteen-like hydrogen bond network with a vacant site suitable for small-molecules recognition and a coordinating K^+^ ion contributing to its stability (for details, see Fig. S10A). Contrarily, the “*closed*” G-triad occurred only in the **2d** structure that was obtained after the transition from 1KF1-like to **2d** through the intermediate **24d**. It was characterized by a specific rearrangement of the Hoogsteen-like hydrogen bonds without any coordinating K^+^ ion.

Most of the G-triads observed in our simulation occurred at the 5′-end, while only one G-triad (**3u**) was observed at the 3′-end (Fig. 2c), with the G-triplet slipping towards the 5′-end direction (*up*). In addition, only two conformations (**24d** and **34d**) exhibited the simultaneous slippage of two G-triplets leaving just two guanine residues at the 5′-end or the 3′-end (Fig. 2e,f, respectively), thus preventing the G-triad formation.

The free energy associated to the vertical slippage of the G-triplets in basin C was calculated using the reweighting algorithm. In such case, the Hb_core_ was used as unbiased CV, which is related to the number of their corresponding Hoogsteen hydrogen bonds, such as Hb_core2d_, Hb_core3d_, Hb_core3u_, Hb_core24d_ and Hb_core34d_ (for details, see Table S2 and Table 2).

**Table 2:** Free energy values with the standard deviation of each slipped conformation found in the basin C, calculated after the reweighting procedure.

Structure	Free Energy^*^ (*kJ/mol*)
**2d**	14.6 ± 3.6
**3d**	16.0 ± 7.7
**3u**	28.2 ± 11.1
**4d**	13.0 ± 5.1
**24d**	34.6 ± 3.6
**34d**	13.0 ± 2.1

The reweighted algorithm led us to better characterize each conformation on the basis of their different free energy value. Specifically, structures **2d**, **3d**, **4d** and **34d** showed the better free energy profile (Table 2), while, among the conformations with one single G-triplet slippage, **3u**, the only characterized by the slippage movement towards the 5′-end direction, owns the highest energetic value and results as the less probable structure. This behaviour could be probably due to the steric hindrance between A1 and G14 residues (Supplementary Information, Figure S15).

In order to verify their geometrical stability, each slipped conformation was submitted to MDs. Interestingly, looking at the progression of the Root Mean Square deviation (RMSd) (Supplementary Information, Figure S11A–F) after 200 ns of MDs, different dynamic behaviours were observed, with only **3d** and **4d** being the most geometrically stable (all details of **24d**, **3d**, **3u**, **2d** and **34d** MDs are described in the Supplementary Information Figure S12–S19). This finding is in agreement with the free energy calculation (Table 2), which retrieved **3d** and **4d** as the most energetically favoured. Given the lowest free energy value of **4d**, we investigated more in details its dynamic behaviour by extending its MDs to 1 µs.

### 4d as the most probable two G-tetrads structure with a well-defined G-triad

**Figure 3:**
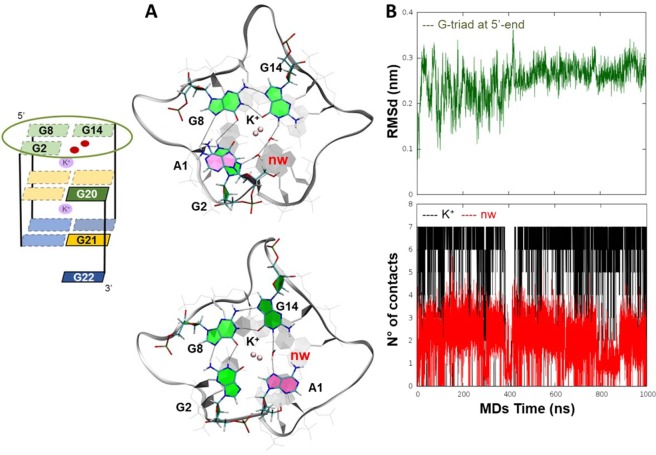
The best geometrical stability of the “open” G-triad of **4d** structure. (**A**) 3D structures (upper panel) of the G-triad (G2:G8:G14) belonging to **4d** conformation, with two water molecules network (nw) (upper panel) and one water bridge between G14 and A1 (lower panel), both miming the G16 slipped residue (**B**) In the upper panel, plot showing the RMSd trend calculated during the MDs on the G-triad at 5′-end, formed by G2:G8:G14 residues (dark-green line). In the lower panel, the plot shows the number of contacts between K^+^ ion and the guanines O6 oxygen atoms (black line) and the number of contacts between bulk water (nw) and the G8 and G20 residues (red line) during the MDs.

1 µs long MDs of **4d** globally revealed a good geometrical stability of its G-tetrads (Supplementary Information, Figure S20A). An increase of the RMSd trend, calculated on all the DNA nucleobases, was observed only in the first 200 ns and was caused by the fluctuations of the first and the third loops, as shown in the Root Mean Square Fluctuation (RMSF) plot (Supplementary Information, Figure S20B). Interestingly, the highest fluctuation was observed for the first adenine nucleobase (A1) at 5′, which changed its orientation from *anti to syn* (for details, see Supplementary Information Movie SM2). This conformational change allowed the stacking of A1 over G20, thus hampering the rearrangement to the native crystallographic structure and stabilizing the two G-tetrad planes. Furthermore, the positioning of A1 on the same plane of the G-triad, together with the water-bridge network among the atoms N7 and H1 of A1 and G14 residues (Fig. 3A, upper panel), concurred to a further stabilization of the G-tetrad. An additional contribution to the stability of the **4d** G-triad is due to the bypiramidal coordination of the K^+^ ion with the O6 atoms of the G-triad and the lower G-tetrad (Fig. 3B, lower plot). Similarly, two water molecules engaged hydrogen bonds with G2:G14 residues, thus miming the slipped G20 residue (Fig. 3A) and favouring a further stability of the G-triad, as clearly emerges after 600 ns of MDs (Fig. 3B, upper plot).

A movie showing the vertical slippage mechanism leading to **4d** conformation can be found in the Supplementary Information Movie SM1.

### Experimental studies

**Figure 4:**
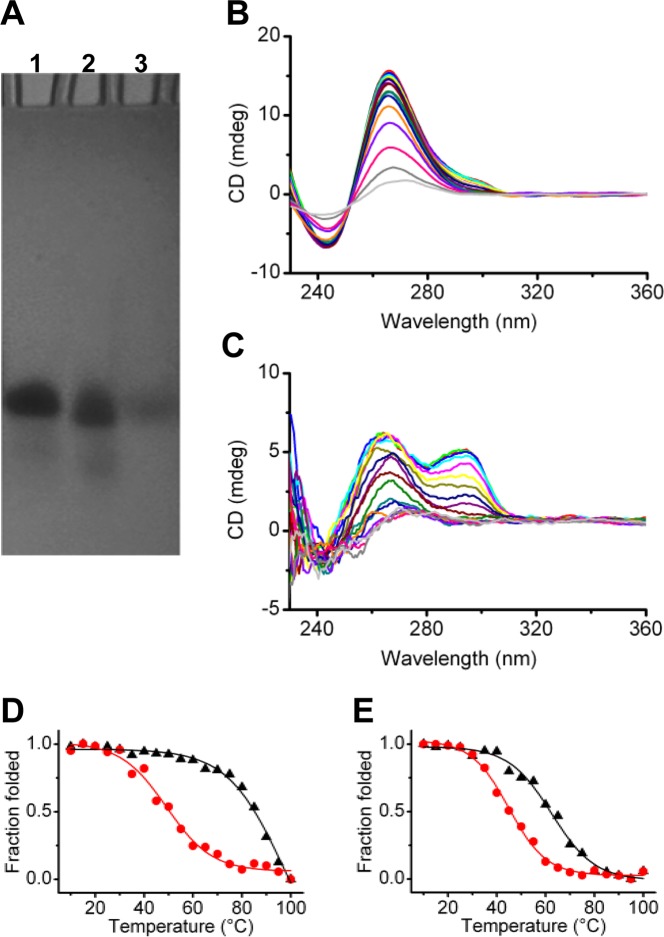
(**A**) Native gel electrophoresis analysis of *h-tel*_23_ (lane **1**) and *h-tel*_*22tr*_ (lane **2**) prepared in 20 mM potassium phosphate buffer (pH 7.0) containing 70 mM KCl, 0.2 mM EDTA, and 40% PEG200. Lane **3**: bromophenol blue. CD spectra of (**B**) *h-tel*_23_ and (**C**) *h-tel*_*22tr*_ in 40% PEG200 containing K^+^-buffer as a function of temperature (from 10 to 100 °C with temperature increase of 5 °C); and normalized CD melting curves of (**D**) *h-tel*_23_ and (**E**) *h-tel*_*22tr*_ obtained by following changes of CD signal at 265 (black) and 295 (red) nm.

Most of the predicted folding intermediates of the parallel telomeric G4 found in the basin C contain one G-triad and two G-tetrad planes. These structures represent transient metastable states, and it is very difficult to demonstrate experimentally their existence. However, to have some experimental information about the possible existence of such a structure in solution, we investigated the construct obtained by removing the last guanine base from the *d*[TAG_3_(TTAGGG)_3_] sequence (*h-tel*_23_), i.e. the 22-nt sequence *d*[TAG_3_(TTAGGG)_2_TTAGG] (hereafter named *h-tel*_*22tr*_). The G4-forming sequence *h-tel*_23_ adopts, in a 40% PEG200 containing K^+^-solution, the same parallel-stranded conformation with three G-tetrad planes found for *h-tel* by X-ray^23^. The removal of a single guanine at the 3′ end of the sequence rules out the possibility of forming a monomolecular G4 structure with three G-tetrads. In order to assess the molecularity of *h-tel*_*22tr*_ in 40% PEG200 containing K^+^-solution, non-denaturing gel electrophoresis analysis was performed by using as reference the *h-tel*_23_ G4 in the same buffer conditions. As shown in Fig. 4A (see also Supplementary Information, Figure S21), *h-tel*_*22tr*_ moves essentially as a single band in the gel, exhibiting a mobility close to *h-tel*_23_. This clearly indicates the absence of multimolecular DNA complexes and suggests that *h-tel*_*22tr*_ adopts a structure (or structures) comparable to that of *h-tel*_23_ under such experimental conditions. Then, a spectroscopic investigation of the species formed upon folding of *h-tel*_*22tr*_ sequence was performed in comparison with *h-tel*_23_ by circular dichroism (CD). Previous studies have demonstrated that the parallel conformation is not the only populated conformation for the telomeric G4 under the PEG-induced crowding conditions, but a non-negligible amount of hybrid conformation coexists with the parallel one^39^. Fig. 4B,C show the CD spectra of *h-tel*_23_ and *h-tel*_*22tr*_ in the PEG-containing buffer, recorded in the range 10–100 °C with temperature increase of 5 °C. In general, the CD bands of DNA molecules are generated when the bases are chirally oriented with respect to each other, i.e. when the DNA is structured. Owing to the heterotypic nature of the two faces of guanines, each G can stack onto the adjacent one through the same or the opposite face, leading to a homopolar or heteropolar stacking, respectively^40^. The former happens when two contiguous Gs have opposite glycosidic bond angle conformation (e.g. *anti*-*syn*), whereas the latter happens when the two Gs have the same glycosidic bond angle (e.g. *anti*-*anti*). The CD spectrum of a G-rich oligonucleotide characterized by a positive band at 265 nm and a negative one at 243 nm is typical of parallel G4 structures having heteropolar stacks. On the other hand, a positive band at 295 nm is characteristic of the homopolar stacks of hybrid/antiparallel G4 conformations. In agreement with the presence of the parallel-stranded G4 topology, *h-tel*_23_ displayed a positive band at 265 nm and a negative one at 243 nm in the spectra (Fig. 4B). However, at low temperatures (up to about 45 °C), the spectra clearly show a shoulder at around 295 nm. This is in agreement with the presence of a certain amount of hybrid/antiparallel conformation(s). In addition, plotting melting curves from the CD data at two wavelengths, i.e. 265 and 295 nm (Fig. 4D), gave two different T_1/2_ values (95 ± 2 and 50 ± 1 °C, respectively), thus suggesting the presence of two different species. CD spectra of *h-tel*_*22tr*_ as a function of temperature show that the shoulder at around 295 nm is much more pronounced in this case, thus indicating that probably the equilibrium between parallel and hybrid/antiparallel species is more shifted toward the latter than in the case of *h-tel*_23_. Plotting melting curves from the *h-tel*_*22tr*_ CD data at 265 and 295 nm (Fig. 4E) gave T_1/2_ values of 63 ± 1 and 45 ± 1 °C, respectively, also suggesting the existence of different species in solution.

## Discussion

The parallel topology of the human telomeric sequence *d*[AG_3_(TTAGGG)_3_] can be considered the G4 conformation with the most probable biological role among all G4 structures^21,32^. Moye *et al*. demonstrated that the telomerase recognizes and binds different parallel G4s with high affinity, whereas the antiparallel conformations are not good substrates, despite their much higher stability^32^. Also HMGB1, an ubiquitous non-histone protein engaged in many DNA events, such as DNA repair, transcription and telomere maintenance, has a preference for the unimolecular parallel TelG4^41^. Currently, although it has been found that the stabilization of G4 structure can affect the telomerase enzyme activity with a resultant antineoplastic effect, the characterization of its folding intermediate structures formed in solution are still elusive at atomistic level^34^. In fact, for the propeller-type conformation, the G-triplex formation was considered as the less probable intermediate state^42^, while the most natural arrangement for such kind of topology is the vertical strand-slippage movement of the G-triplets that leads to two G-tetrad planes and one G-triad, as demonstrated by standard MDs^30^.

In several instances, Metadynamics-based approaches allowed a detailed atomistic description of meaningful metastable folded states^43–47^, supporting, in some cases, even their characterization by means of experimental techniques^48,49^. In this work, using WT-MetaD simulations, we have provided a comprehensive picture of all the possible ensemble conformations emerging during the folding process of the 22-nt parallel topology G4 in solution, which have never been detected yet, at the best of our knowledge. In addition, we also have employed an innovative CV (π-π_core_) to investigate the π-π stacking behavior among all *anti*-guanines of the G-tetrads, besides the Hoogsteen hydrogen bonds network of the G-tetrads CVs. By reweighting the WT-MetaD^37^ sampling, we were able to retrieve an ensemble of structures (basin C) having two G-tetrad planes in equilibrium with each other. Thus, our studies enforce the hypothesis that late/early stages of parallel stem formation/disruption may involve cation-stabilized four-stranded intermediates with various degrees of strand slippage and incomplete numbers of tetrads^30^. These results also support the formation mechanism suggested by Stefl *et al*. in 2003^38^, according to which the parallel G4 has clear strand slippage capabilities. In agreement with the models reported in the previous literature, the formation of G-triplex intermediates^28,30^ wasn’t directly detected, while a slowly rearrangement of the slipped strands into the native four-tetrad stem, also consistent with experimental data^21^, was clearly observed and described in this work. This mechanism leads to the formation of a G-triad plane, which can arrange in an “open” and “closed” form based on the G-triplet involved in the vertical slippage^38^. The “closed” G-triad, which exhibited a good geometrical stability, was found only in **2d** conformation and it is reasonably due to the absence of the coordinating K^+^ ion within the G-triad. On the other hand, the “open” conformation of the G-triad was stabilized by a water molecules network established in the vacant site of the slipped guanine, similarly to the NMR structure described by Heddi and co-workers, suitable as possible ligand binding site^35^. However, in the basin C, G4 conformations without a G-triad were also collected and they derived from the simultaneous vertical slippage of two G-triplets. In particular, our WT-MetaD run highlighted that the vertical slippage of two adjacent G-triplets are more probable than the simultaneous movement of two opposed G-triplets. Indeed, the better geometrical stability and probability of existence of **34d** is mainly due to the presence of two Hoogsteen pairs of guanines, able to coordinate K^+^ ions with their O6 oxygens.

Focusing on the directionality of the vertical slippage mechanism, the movement towards the 3′-end position has been found out in almost all the structures collected in the basin C, leading to the observation that the presence of residue A1 at the 5′-end involves a steric hindrance such as to hinder the G-triplets movement in this direction. Therefore, only one structure having 5′-end slippage was found in the basin C, showing also a reduced probability of existence. Its dynamic behavior points out the highest stability of its “open” G-triad coupled to wide fluctuations of the third loop and consequently of the 3′-end position, resulting in agreement with the WT-MetaD as the portion with the most favorable capability for the vertical slippage movement. Going along with these findings, **34d** and **4d**, the two most probable structures found in basin C, showed the vertical slippage of the G-triplets in the 3′-end portion and can be hypothesized as the two most important cation-stabilized four-stranded intermediates occurring during the late/early stages of parallel stem formation/disruption. In particular, **4d** structure is characterized by an “open” G-triad with the best geometrical stability between those found at the 5′-end position.

The whole set of experimental data suggests that the construct obtained by removing the last G from the *d*[TAG_3_(TTAGGG)_3_] sequence forms monomolecular G4-like structures under molecular crowding conditions. Since the removal of the last guanine of the sequence ruled out the possibility of forming G4 structures with three G-tetrads, these structures are supposed to be stabilized by no more than two G-tetrads. Indeed, as expected, they result to be thermally less stable than *h-tel*_23_ structures. However, as for *h-tel*_*22tr*_ parallel structure, we observed a T_1/2_ value of 63 °C, which, compared with that of a two-tetrads-G4 under the same experimental conditions (T_1/2_ = 58.7 °C)^47^, suggests the presence of additional intramolecular interactions that may arise from an additional G-triad. These results are consistent with the hypothesis that G4s containing two G-tetrads and one G-triad may actually be intermediate in the folding of the parallel telomeric G4.

Since a stable G4 structure of a synthetic sequence containing two G-tetrads and one G-triad has already been determined by means of NMR technique^30^, we can enforce the hypothesis on the existence of the ensemble of conformations found in the basin C, which in any case results very difficult to experimentally demonstrate for their structural heterogeneity. Indeed, in literature it is widely reported the difficulty to obtain the parallel propeller-type G4 structure in diluted solution by means of the NMR technique^18^. The concept of forming G4s with missing guanines in the G-tetrads as result of the vertical G-triplets slippage movement enriches and extends the current knowledge of the G4 structures. The vacant site in the “open” G-triads can provide a potential binding site for small-molecule and metabolites recognition^50^, playing a regulatory role in the telomeres elongation and/or in the gene expression and assuming a behavior similar to RNA riboswitches. In fact, several RNA riboswitches are able to bind metabolites, as guanine and guanine derivatives, with associated regulator effect on gene expression^51–53^.

This study aims also to present an alternative computational protocol able to investigate the phase space of other parallel G4 structures, such as the oncogene promoter ones, in order to describe the late/early stages of parallel stem formation/disruption. The discovery of possible four-stranded intermediates with various degrees of strand slippage and incomplete numbers of tetrads for the oncogene promoter G4s would open new scenarios in understanding the process behind the regulation of gene expression.

## Methods

### System setup and MDs

The crystal structure of the parallel stranded G4 with the 22-nt human telomeric sequence *d*[AG_3_(T_2_AG_3_)_3_] and a resolution of 2.1 Å (PDB ID: 1KF1), was used as starting conformation for the computational simulations^17^. K^+^ ions coordinating the G-tetrad O6 atoms were retained, while all the crystallized water molecules were removed.

MD simulations were carried out with the GROMACS code ver.4.5.1^54^ implemented with the PLUMED plugin 1.3^55^ to perform the WT-MetaD sampling.

The G4 nucleic acid was treated using the standard *parm99* Amber force field modified with the recently developed parmbsc0^56,57^, in combination with the recently-developed corrections ε/ζOL1 and χOL4 to improve the description respectively of the ε/ζ and χ G-quadruplex torsions^58–63^.

The topology file of the system was generated using the xleap module of the AmberTools program^64^ and converted in a suitable GROMACS file format using the Acpype script^65^.

G4 nucleic acid was solvated in a truncated dodecahedron box with TIP3P^66^ water solvent model using periodic boundary conditions and it was neutralized by adding 19 K^+^ ions. Long-range electrostatic interactions were treated using Particle-Mesh Ewald (PME)^67,68^ with a cut-off of 1.0 nm. Van der Waals (VdW) interactions were calculated using also a cut-off of 1.0 nm. All bonds were dealt with the LINCS algorithm^69^.

Firstly, the whole system was subjected to a minimization phase using 5000 steps with the steepest descent algorithm to resolve bad steric contacts and then equilibrated under constant pressure and temperature at 1 atm and 300 K for 4 ns using a time step of 2 fs. For the temperature control the V-rescale algorithm has been used^70^, while the Parrinello-Rahman^71^ barostat to monitor the pressure. In order to verify the stability of the system, 200-ns-long MDs at room temperature were performed in the NVT ensemble.

### Well-tempered metadynamics simulation (WT-MetaD)

To obtain an exhaustively local conformational exploration of the parallel G4, we applied the Well-Tempered variant of Metadynamics (WT-MetaD)^33^. Initial Gaussians height (W) was set at 2.48 kJ/mol with a deposition rate equal to 1 ps. The bias factor was fixed to 20. Two collective variables (CVs) were used to sample local conformational space of the parallel G4: the Coordination CV to describe the native Hoogsteen hydrogen bonds (Hb_core_ 1KF1) and the number of stacking interactions among the guanines of each G-triplet (π-π_core_) (for more details see *Hoogsteen Hydrogen bonds (Hb*_*core*_) and *π-π*_*core*_ *CV* paragraphs in the Supplementary Information, including Figure S1). The first CV was evaluated considering the total number of contacts between the acceptor atoms of the G-tetrad, listed in group G1, and the donor ones, put in group G2 (see Table S1 in Supplementary Information), using a Gaussian width of 0.2. For the second CV, since the lack of a proper CV able to describe such π-π interactions, we have referred to the aromatic packing density which characterizes the environment of a typical G-core likewise has been already addressed by Giberti F. *et al*.^36^ for the nucleation of urea crystals. For a better clarity, the function of π-π_core_ is summarized in the Supplementary Information. A Gaussian width of 0.5 was used for this CV. It is worth to underline that, in order to verify the slippage mechanism involving only the G-tetrads, during the WT-MetaD, a complete unfolding of G4 DNA was prevented by using a potential restraint able to focus the conformational sampling only on those structure having more than five Hb_core_ 1KF1 and eight π-π_core_ CVs (for more details see *Potential Restraint* paragraph in the Supplementary Information).

### The reweighted FES

Once the free energy of the WT-MetaD simulation is converged, the FES was reconstructed using the reweighting algorithm^37^ (Fig. 2) as function of two unbiased CVs: the first CV (named as “RMSd_*(G-core)*_ *vs* 1KF1_*Cry*_”) corresponds to the RMSd of the G-tetrads superimposed to those of the 1KF1 crystallographic model (the latter hereafter named as 1KF1_*Cry*_); as second CV we used a Coordination to describe the number of Hoogsteen hydrogen bonds (Hb_core_) formed in the G-tetrads. In particular, as concern the second CV, we defined exactly the number of Hoogsteen hydrogen bonds for each structure having two G-tetrad planes as Hb_core2d_, Hb_core3d_, Hb_core3u_, Hb_core24d_ and Hb_core34d_ (see Table S2 in Supplementary Information) with respect to 1KF1_*Cry*_ (see Table S1 in Supplementary Information). The proper Hb_core_ value of each structure was assigned following the flow diagram reported in Figure S22.

### MDs on G4 conformations representing the basin C

The stability of the slipped G4 conformations found in basin C was assessed calculating the RMSd during 200 ns long MDs, by applying the same protocol previously described for the starting 1KF1_*Cry*_. Finally, MDs of **4d** structure was extended to 1 µs MDs, in order to better analyze the most stable slipped structure found in basin C.

### DNA sample preparation

The *d*[TAG_3_(TTAGGG)_3_] (*h-tel*_23_) and *d*[TAG_3_(TTAGGG)_2_TTAGG] (*h-tel*_*22tr*_) sequences were synthesized and purified as already described^72^. The oligonucleotides were dissolved in 20 mM potassium phosphate buffer (pH 7.0) containing 70 mM KCl, 0.2 mM EDTA, and 40% PEG200. The concentration of oligonucleotides was determined by UV adsorption measurements at 95 °C using appropriate molar extinction coefficient values ε (λ = 260 nm) calculated by the nearest-neighbor model^73^. Each sample was heated to 95 °C for 5 min, gradually cooled to room temperature overnight, and then incubated for at least 24 h at 4 °C before data acquisition.

### Gel electrophoresis

Native gel electrophoresis analysis was carried out on 15% polyacrylamide gel at 25 °C, which was run at 80 V for 4 h in 1 × TB buffer (pH 7.5) supplemented with 10 mM KCl. A concentration of 50 μM was used for the oligonucleotides, which were prepared in a 40% PEG200 containing K^+^-buffer. Prior to loading the mixture onto the gel, 1 μL of glycerol solution (60% v/v) was added. The total volume loaded in each well was 10 μL. Gel was imaged by UV-shadowing, using the exposure wavelength of 254 nm for nucleobase detection.

### Circular dichroism spectroscopy

CD experiments were recorded on a Jasco J-815 spectropolarimeter equipped with a PTC-423S temperature controller. Spectra were recorded in a quartz cuvette with 1 mm path length in the 220–360 nm wavelength range and averaged over three scans. The scan rate was set to 100 nm/min, with a 1 s response time and 1 nm bandwidth. Buffer baseline was subtracted from each spectrum. Experiments were performed using 10 μM oligonucleotide concentration. CD melting experiments were carried out by collecting data in the range 10–100 °C using a temperature step of 5 °C. The samples were incubated at each temperature for a suitable time to achieve the equilibrium. Reaching equilibrium at each temperature was guaranteed by the achievement of superimposable CD spectra on changing time. The CD melting curves were obtained by following changes of CD signal at 265 and 295 nm. Apparent melting temperatures (T_1/2_) were determined from curve fit using Origin 7.0 software^74^.

## Supplementary information

Supplementary Information.
