# Supplementary material for: Folding intermediate states of the parallel human telomeric G-quadruplex DNA explored using Well-Tempered Metadynamics
Source: Sci Rep. 2020 Feb 21;10:3176. doi: 10.1038/s41598-020-59774-x (PMC7035250; doi:10.1038/s41598-020-59774-x)
Supplement: Supplementary file 1 — Supplementary Information. [file 41598_2020_59774_MOESM1_ESM.docx]

**Supplementary Information for**

**Folding intermediate states of the parallel human telomeric G-quadruplex DNA explored using Well-Tempered Metadynamics**

*Roberta Rocca,^1,2^ Ferruccio Palazzesi,^3^ Jussara Amato,^4^ Giosuè Costa,^1,2^ Francesco Ortuso,^1,2^ Bruno Pagano,^4^ Antonio Randazzo,^4^ Ettore Novellino,^4^ Stefano Alcaro,^1,2^ Federica Moraca,^1,2,4*^ Anna Artese^1,2,*^*

*^1^ Dipartimento di Scienze della Salute, Università “Magna Græcia” di Catanzaro, Campus Salvatore Venuta, Viale Europa, 88100 Catanzaro, Italy.*

*^2^ Net4Science srl, Università "Magna Græcia" di Catanzaro, Campus Salvatore Venuta, Viale Europa, 88100, Catanzaro, Italy.*

*^3^ Research Informatics, Computational Chemistry & Cheminformatics, Aptuit an Evotec Company, Via A. Fleming 4, 37135 Verona (Italy).*

*^4^ Department of Pharmacy, University of Naples "Federico II", Via D. Montesano 49, Naples 80131, Italy.*

*^*^Corresponding Authors: artese@unicz.it; federica.moraca@unina.it*

^*^Email: artese@unicz.it; Phone: +39 0961 369 4297; Fax: +39 0961 369 4073;

^*^Email: federica.moraca@unina.it; Phone: +39 081 678551.

**π-π_core_ CV**

The π-π_core_ CV was described with a modified version of the crystallinity (S) CV, previously developed by Giberti *et al.* as a sum of individual molecular contributions, in order to study the nucleation of urea crystals.^1^ Indeed, the guanines stacking is linked to the packing density of the molecular environment surrounding molecule *i* and to the relative orientation of molecule *i* with respect to its neighbours. The final expression for the CV S is:


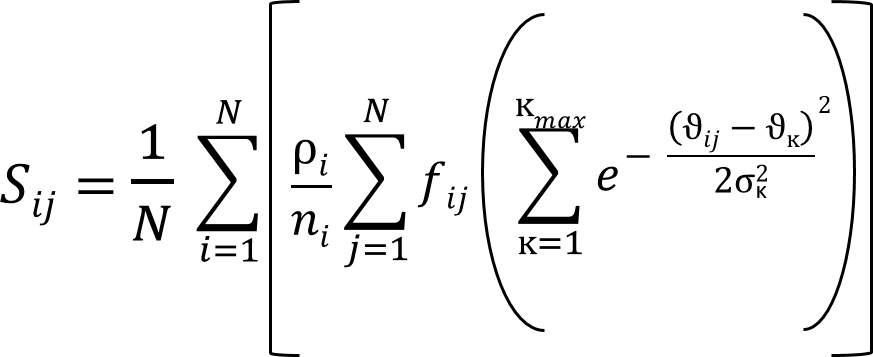


S can be physically interpreted as the fraction of molecules locally ordered according to the series of reference angle ὐ_k_, that represents the right orientation for the stacking. In particular, we considered the angle between the vectors N1-N7 of two consecutive guanines, as reported in Figure S1. Therefore, if one guanine is surrounded by *n_i_* guanines all orientated according to the angle ὐ_k_, the CV value will be 1 and the stacking interaction will be taken into account. Contrarily, if the orientation of its neighbours is randomly distributed, the orientation-dependent term becomes small and so the CV will assume a value near zero. In all other cases, S will have an intermediate value between 0 and 1.

To consider all stacking interactions among the guanines core, as single overall value, we used the Polynomial Combination of CVs, given by the functional form:


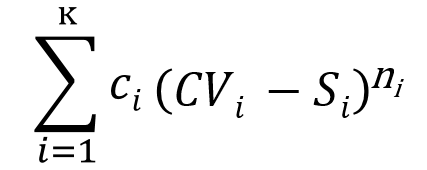


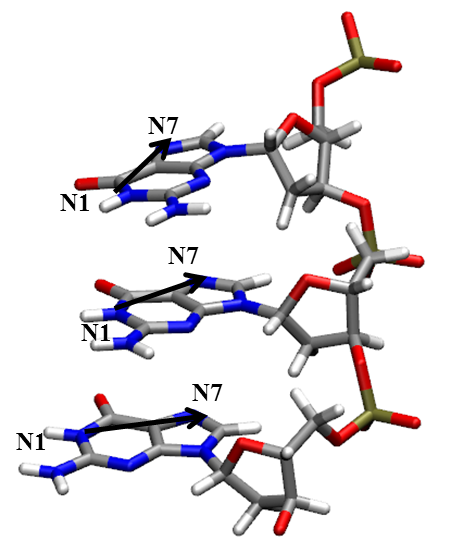


**Figure S1.** 3D representation of vectors N1-N7 of consecutive guanines considered for the definition of the angles ὐ_k_.

**Hoogsteen Hydrogen bonds (Hb_core_) CV**

The Hoogsteen hydrogen bonds were described with the coordination CV (COORD), which is implemented as the sum:


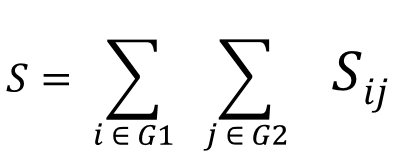


where this sum is extended to all pairs of atoms with *i* ϵ ***G*_1_** and *j* ϵ ***G*_2_**. The individual contributions *S_ij_* are defined using a switching function that, in the present case, is given by:


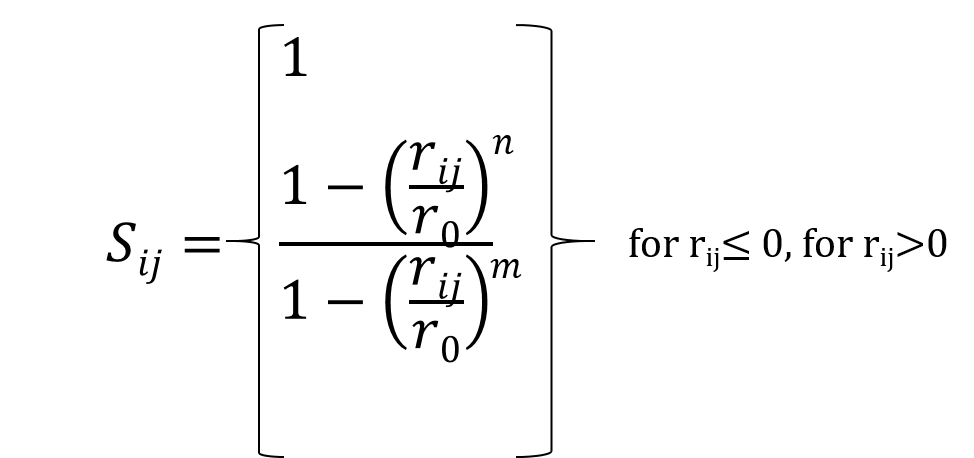


where r*ij* = |r*i –* r*j*| - d*_0_* and *i* and *j* are the donor and acceptor hydrogen bond atoms of the guanines used to calculate the number of hydrogen bonds, respectively. In order to treat the atoms in a pairwise fashion, the PAIR keyword was also used, defining the specific bonds established between the two groups to be monitored.

**Table S1:** List of the atom pairs used to define the Hb_core_ 1KF1 CV.

| ***Hb****_core 1KF1_* | **H-bond acceptor** | **H-bond donor** |
| --- | --- | --- |
|  | O6 (G2) | H1 (G20) |
|  | N7 (G2) | H21 (G20) |
|  | O6 (G8) | H1 (G2) |
|  | N7 (G8) | H1 (G2) |
|  | O6 (G14) | H1 (G8) |
|  | N7 (G14) | H21 (G8) |
|  | O6 (G20) | H1 (G14) |
|  | N7 (G20) | H21 (G14) |
|  | O6 (G3) | H1 (G21) |
|  | N7 (G3) | H21 (G21) |
|  | O6 (G9) | H1 (G3) |
|  | N7 (G9) | H1 (G3) |
|  | O6 (G15) | H1 (G9) |
|  | N7 (G15) | H21 (G9) |
|  | O6 (G21) | H1 (G15) |
|  | N7 (G21) | H21 (G15) |
|  | O6 (G4) | H1 (G22) |
|  | N7 (G4) | H21 (G22) |
|  | O6 (G10) | H1 (G4) |
|  | N7 (G10) | H1 (G4) |
|  | O6 (G16) | H1 (G10) |
|  | N7 (G16) | H21 (G10) |
|  | O6 (G22) | H1 (G16) |
|  | N7 (G22) | H21 (G16) |

**Table S2:** List of the pair atoms defining the native Hbonds of each structure with two G-tetrads core denominated Hb_core 2d_, Hb_core 3d,_ Hb_core 3u_, Hb_core 24d_ and Hb_core 34d_.

| ***Hb****_core 2d_* | **H-bond acceptor** | **H-bond donor** |  |
| --- | --- | --- | --- |
|  | O6 (G3) | H1 (G21) |  |
|  | N7 (G3) | H21 (G21) |  |
|  | O6 (G8) | H1 (G3) |  |
|  | N7 (G8) | H1 (G3) |  |
|  | O6 (G15) | H1 (G8) |  |
|  | N7 (G15) | H21 (G8) |  |
|  | O6 (G21) | H1 (G15) |  |
|  | N7 (G21) | H21 (G15) |  |
|  | O6 (G4) | H1 (G22) |  |
|  | N7 (G4) | H21 (G22) |  |
|  | O6 (G9) | H1 (G4) |  |
|  | N7 (G9) | H1 (G4) |  |
|  | O6 (G16) | H1 (G9) |  |
|  | N7 (G16) | H21 (G9) |  |
|  | O6 (G22) | H1 (G16) |  |
|  | N7 (G22) | H21 (G16) |  |
|  | O6 (G3) | H1 (G21) |  |
|  | N7 (G3) | H21 (G21) |  |
|  | O6 (G9) | H1 (G3) |  |
|  | N7 (G9) | H1 (G3) |  |
|  | O6 (G14) | H1 (G9) |  |
|  | N7 (G14) | H21 (G9) |  |
|  | O6 (G21) | H1 (G14) |  |
|  | N7 (G21) | H21 (G14) |  |
|  | O6 (G4) | H1 (G22) |  |
| ***Hb****_core 3d_* | N7 (G4) | H21 (G22) |  |
|  | O6 (G10) | H1 (G4) |  |
|  | N7 (G10) | H1 (G4) |  |
|  | O6 (G15) | H1 (G10) |  |
|  | N7 (G15) | H21 (G10) |  |
|  | O6 (G22) | H1 (G15) |  |
|  | N7 (G22) | H21 (G15) |  |
|  | O6 (G2) | H1 (G20) |  |
|  | N7 (G2) | H21 (G20) |  |
|  | O6 (G8) | H1 (G2) |  |
|  | N7 (G8) | H1 (G2) |  |
|  | O6 (G15) | H1 (G8) |  |
|  | N7 (G15) | H21 (G8) |  |
|  | O6 (G20) | H1 (G15) |  |
|  | N7 (G20) | H21 (G15) |  |
|  | O6 (G3) | H1 (G21) |  |
| ***Hb****_core 3u_* | N7 (G3) | H21 (G21) |  |
|  | O6 (G9) | H1 (G3) |  |
|  | N7 (G9) | H1 (G3) |  |
|  | O6 (G16) | H1 (G9) |  |
|  | N7 (G16) | H21 (G9) |  |
|  | O6 (G21) | H1 (G14) |  |
|  | N7 (G21) | H21 (G16) |  |
|  | O6 (G3) | H1 (G20) |  |
|  | N7 (G3) | H21 (G20) |  |
|  | O6 (G9) | H1 (G3) |  |
|  | N7 (G9) | H1 (G3) |  |
|  | O6 (G15) | H1 (G9) |  |
|  | N7 (G15) | H21 (G9) |  |
|  | O6 (G20) | H1 (G15) |  |
|  | N7 (G20) | H21 (G15) |  |
|  | O6 (G4) | H1 (G21) |  |
| ***Hb****_core 4d_* | N7 (G4) | H21 (G21) |  |
|  | O6 (G10) | H1 (G4) |  |
|  | N7 (G10) | H1 (G4) |  |
|  | O6 (G16) | H1 (G10) |  |
|  | N7 (G16) | H21 (G10) |  |
|  | O6 (G21) | H1 (G16) |  |
|  | N7 (G21) | H21 (G16) |  |
| ***Hb****_core 24d_* | O6 (G3) | H1 (G20) |  |
|  | N7 (G3) | H21 (G20) |  |
|  | O6 (G8) | H1 (G3) |  |
|  | N7 (G8) | H1 (G3) |  |
|  | O6 (G15) | H1 (G8) |  |
|  | N7 (G15) | H21 (G8) |  |
|  | O6 (G20) | H1 (G15) |  |
|  | N7 (G20) | H21 (G15) |  |
|  | O6 (G4) | H1 (G21) |  |
|  | N7 (G4) | H21 (G21) |  |
|  | O6 (G9) | H1 (G4) |  |
|  | N7 (G9) | H1 (G4) |  |
|  | O6 (G16) | H1 (G9) |  |
|  | N7 (G16) | H21 (G9) |  |
|  | O6 (G21) | H1 (G16) |  |
|  | N7 (G21) | H21 (G16) |  |
|  | O6 (G3) | H1 (G20) |  |
|  | N7 (G3) | H21 (G20) |  |
|  | O6 (G9) | H1 (G3) |  |
|  | N7 (G9) | H1 (G3) |  |
|  | O6 (G14) | H1 (G9) |  |
|  | N7 (G14) | H21 (G9) |  |
|  | O6 (G20) | H1 (G14) |  |
|  | N7 (G20) | H21 (G14) |  |
|  | O6 (G4) | H1 (G21) |  |
| ***Hb****_core 4d_* | N7 (G4) | H21 (G21) |  |
|  | O6 (G10) | H1 (G4) |  |
|  | N7 (G10) | H1 (G4) |  |
|  | O6 (G15) | H1 (G10) |  |
|  | N7 (G15) | H21 (G10) |  |
|  | O6 (G21) | H1 (G15) |  |
|  | N7 (G21) | H21 (G15) |  |

**
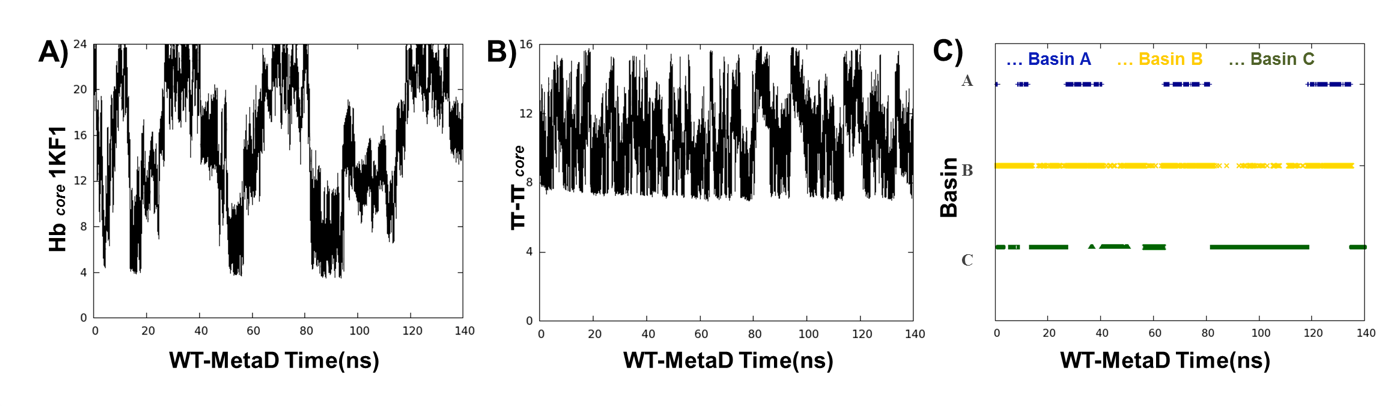
**

**Figure S2: A-B)** Plots showing the phase space represented as (**A**) the Hb *core* 1KF1 and (**B**) π-π *core* CV, explored during the Well-Tempered Metadynamics (WT-MetaD). **C)** Plot showing the recrossing events among the three basins during the WT-MetaD simulation.

**
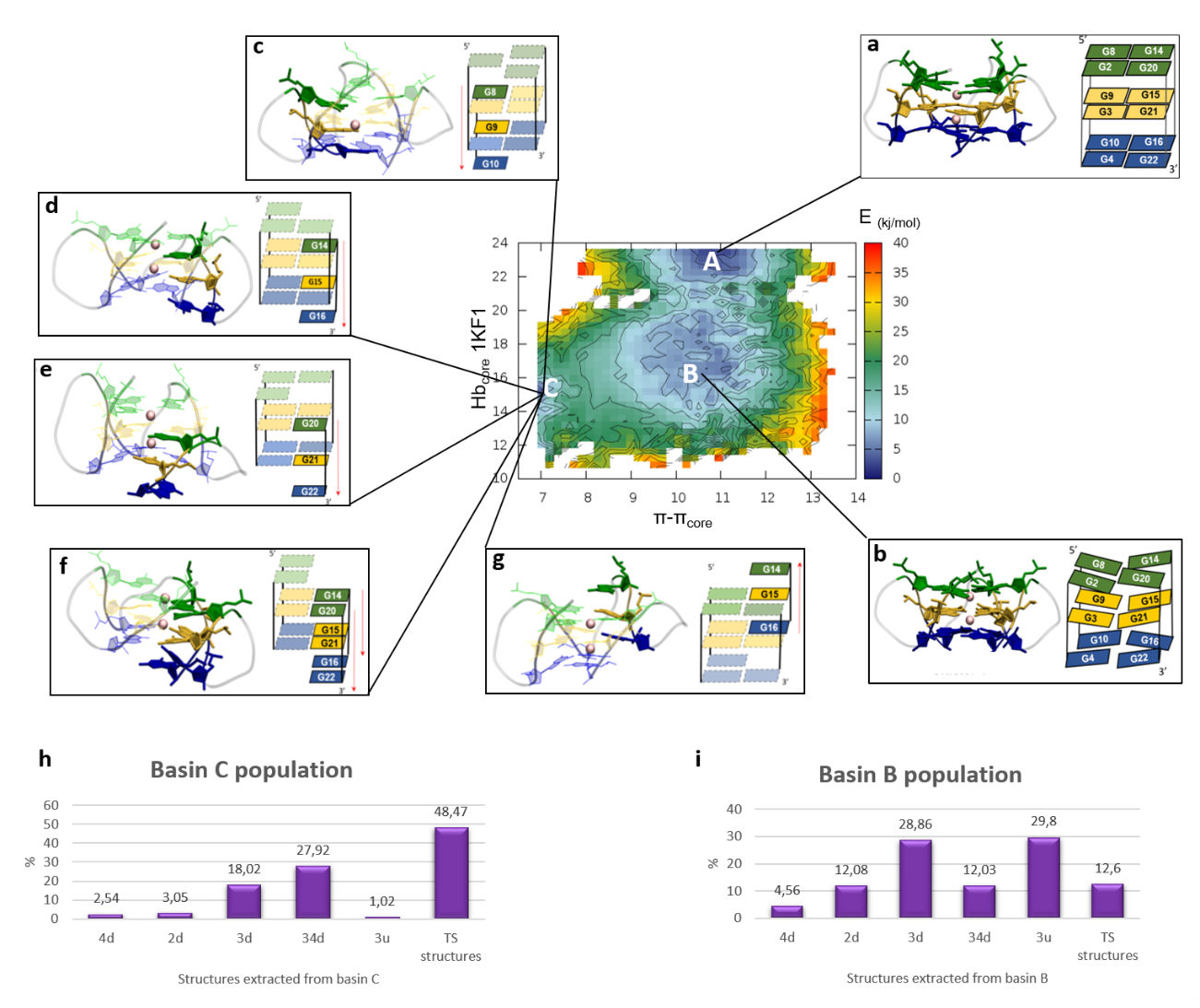
**

**Figure S3.** Free Energy Surface (FES) calculated from the two biased CVs (Hb_core_ 1KF1 and π-π_core_). Three main basins were detected: basin A, characterized with full G-tetrad planes structures (as in the experimental model PDB ID: 1KF1) (Figure S3a); basins B and C characterized by a partial overlap between transition-state structures (TS-structures) (Figure S3b) and slipped structures ensembles: 2d (Figure S3c), 3d (Figure S3d), 4d (Figure S3e), 34d (Figure S3f) and 3u (Figure S3g). In the plots of Figure S3h and Figure S3i is reported the percentage of population of each structure found in basins C and B, respectively.

**The native structure of the parallel G4 in the basin A from the reweighted FES**

The basin A is the energetically deepest one, characterized by an ensemble of structures related to the 1KF1_Cry_, holding three G-tetrads planes perfectly stacking on each other (Figure S1 C-D) and two coordinating bipyramidal antiprismatic K^+^ ions in the center of the G-tetrads. The global number of Hb_core_ and stacking interactions (π-π_core_) ranges from 22 to 24 and from 14 to 16, respectively, thus confirming the high similarity of these ensemble structures with 1KF1*_Cry_* characterized by 24 Hb_core_ and 16 π-π_core_ (see Table S1 and Figure S1 B-C).

As concern the TTA loops, the RMSd matrix (Figure S1A) showed that they are, instead, represented by an ensemble of different transient geometries. Despite the rigidity of the G-tetrads, we observed, in fact, that TTA loop bases can form base-pairing alignments with the guanine residues of the G-tetrad as it has been previously reported by Islam B. *et al*.^2^ In particular, thymine nucleobases T5, T11 and T17 are involved in the formation of pentad, hexad and even heptad planes engaging hydrogen bonds with the guanines of the middle G-tetrad (Figure S4).

Analysing the structures found in the basin A, we observed a wide number of conformations, in which T11 residue interacts with the guanine G9 (Figures S2.B), assuming a planar position with respect the central tetrad such as to form a pentad plane. On the contrary, the thymine nucleobases T5 and T17 are less frequently involved in the formation of hydrogen bonds with the guanines of the central tetrad to achieve hexad or heptad planes (Figure S2 A-C).


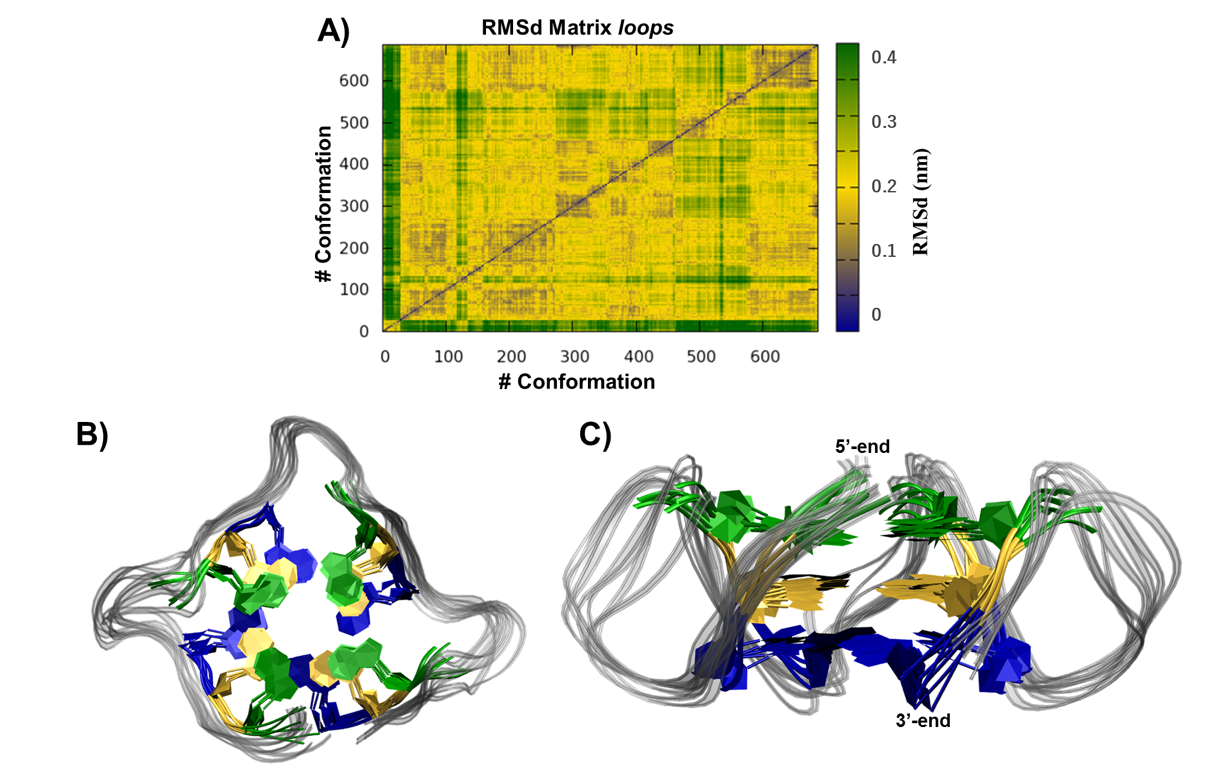


**Figure** **S4**. **A)** Conformational analysis of the conformations found in the basin A assessed by pairwise RMSd matrices, calculated on TTA loops. The minimum and maximum were assigned to a linear colour bar to indicate the variation in RMSd. The high RMSd (green stripes) in RMSd matrix a showed a higher heterogeneity of the TTA loops. In panels **B)** and **C)** the superposition of ten random structures taken from the basin A are reported in top and lateral view, respectively.


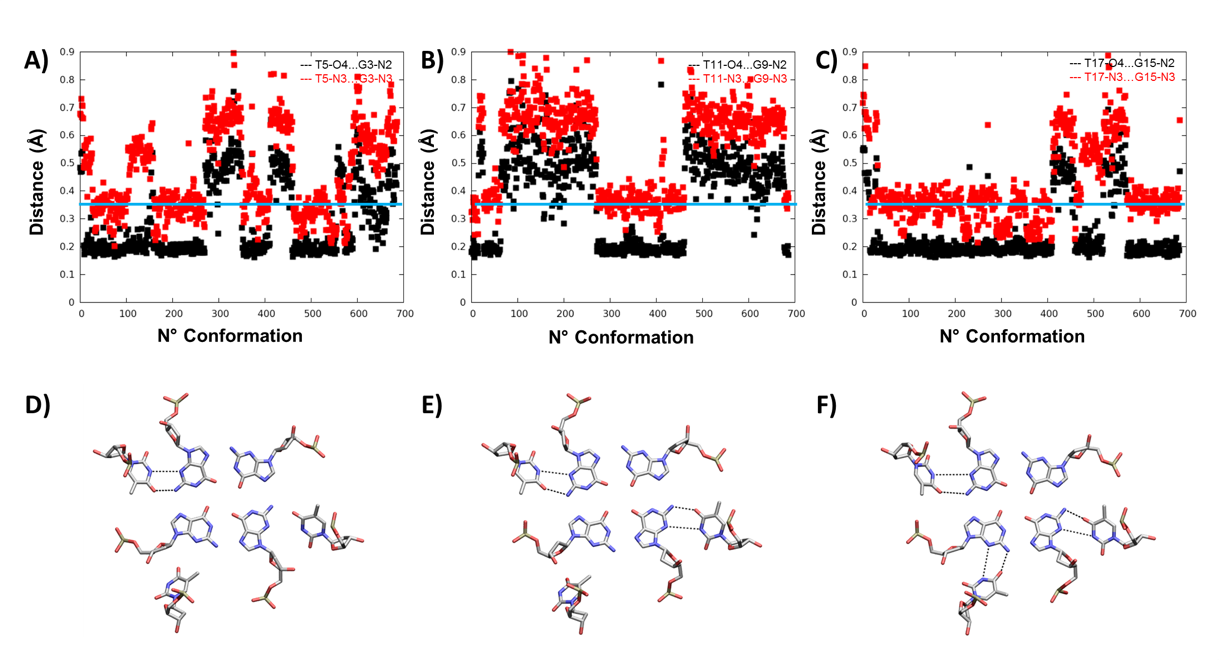


**Figure** **S5.** Plots showing the distribution of the distances between the residues: **A)** T5:G3, **B)** T11:G9 and **C)** T17: G15. (**D**) 3D representation of the pentad with a middle quartet formed by T5 interaction through its Watson–Crick face via shared hydrogen bonds with G3. (**E-F**) Concurrent alignment of T5, T11 and/or 17 with the middle quartet through Watson–Crick face forms an hexad or an heptad, respectively.

**A partial opening of the G-tetrads in the Basin B structures from the reweighted FES**

The basin B, characterized by barely stacked G-tetrad planes, is slightly energetically higher than the basin A. This very low energetically difference can be justified by the significant similarity between the conformations found in these two basins, as highlighted by the average radius of gyration (*R_gyr_*), equal to 0.35 ± 0.01 nm for the basin A and 0.37 ± 0.02 nm for the basin B conformations (see Table S3). Nonetheless, in the basin B, a partial loss of the planarity between the G-tetrad planes is observed, as reflected by the reduced number of the Hb*_core_* interactions (Figure S4), mainly due to a partial opening of the G-tetrads, as detected in the conformations found in the fifth, sixth and seventh clusters (Figure S4B). Unlike all the others, the fifth and sixth clusters showed only one K^+^ ion in the central channel with a corresponding reduced stabilization of G-tetrads and an increase value of the radius of gyration (R*_gyr_*). This event probably represents the first step for the subsequent vertical movement of the guanine triplets.

**Table S3:** R_gyr_ value and number of Hb_core_ calculated for the X-ray model 1KF1 (1KF1*_cry_*) compared to the average R_gyr_ values and numbers of Hb_core_ of the structures found in the basin A and B, with the corresponding standard deviation.

| **G4 structures** | **R*_gyr_* _(nm)_** | **Hb*_core_*** |
| --- | --- | --- |
| **1KF1_cry_** | 0.35 ± 0.00 | 24 ± 0.00 |
| **Basin A** | 0.35 ± 0.01 | 23.71 ± 0.44 |
| **Basin B** | 0.37 ± 0.02 | 19.9 ± 3.84 |


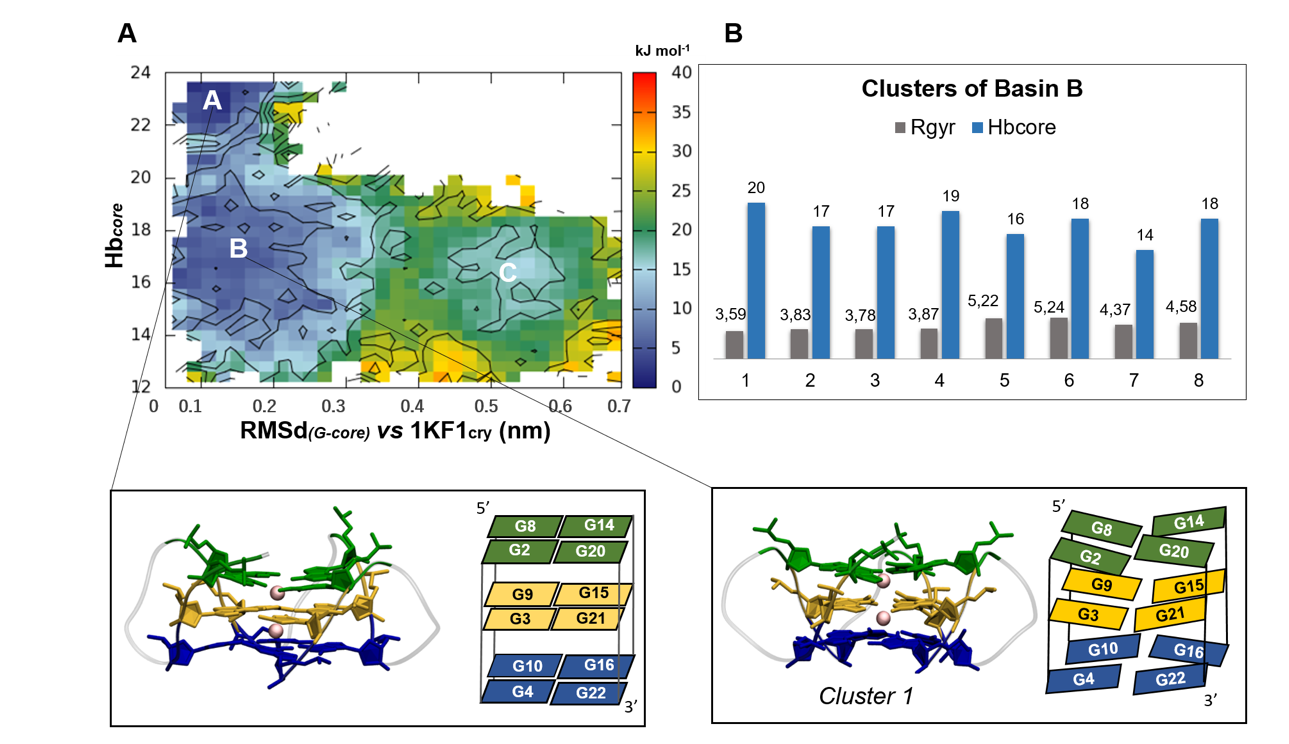


**Figure S6.** The reweighted free energy surface (FES) for the telomeric sequence of G4 in the parallel-propeller topology. (**A**) The reweighted FES with three main energy minima: the energetically deepest one is the basin A, which represents an ensemble of conformations very similar to the 1KF1*_Cry_* with three well-structured G-tetrad planes. The second one, the basin B, with an ensemble of G4 structures characterized by a partial distancing of the guanine residues in the G-tetrad planes. (**B**) Histogram plot showing the radius of gyration (R_gyr_) values of the G-tetrads and the number of Hb*_core_* for the most representative structure of the eight clusters found in the basin B.

**Convergence of the WT-MetaD sampling**

The convergence of the WT-MetaD simulation was tested by checking if the two biased CVs sampled all the possible permitted values, allowing the system to reversibly evolve back and forth several times in the relevant configurational space (see Figures S7 A-B). These recrossing events were a good sign of a well-chosen set of CVs. However, to provide a picture of the free energy convergence, the time evolution of the free energy difference between the three basins was estimated during the 140 ns of WT-MetaD. As reported in Figure S7 A-C, after 100 ns, the free energy converged. The CVs intervals used to identify the three energetic minima are also reported in the caption of Figure S7. Finally, a further evidence of the convergence can be observed from the plot of the Gaussian heights (W) added to the system (Figure S8), which are close to zero after 140 ns of WT-MetaD.^3^


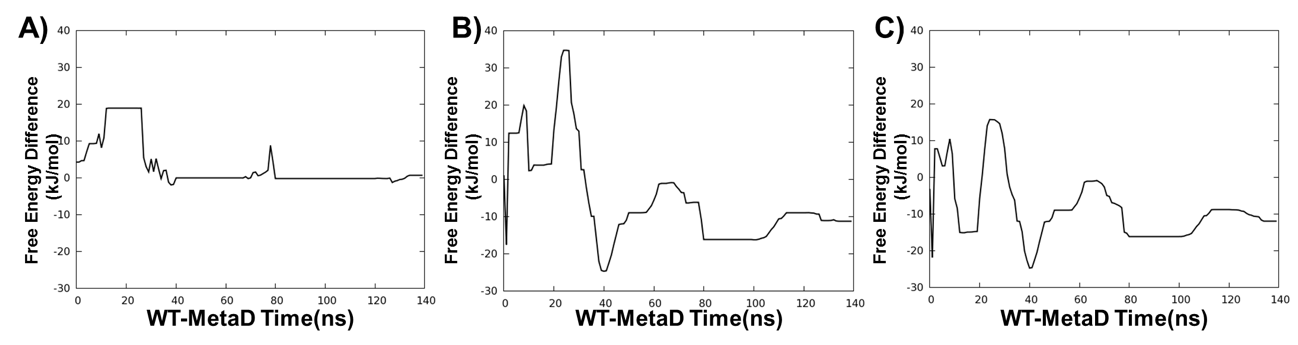


**Figure S7:** The free-energy difference between the three main minima, achieved after 140 ns of WT-MetaD. In particular, the panels **A)**, **B)** and **C)** show the free energy differences between basins A and B, A and C, B and C, respectively, as a function of the simulation time. In particular, the phase spaces corresponding to each basin were defined by the following intervals of the two unbiased CVs: 22<Hb*_core_*<24 and 0.10<RMSd<0.15 nm, for the basin A; 16<Hb*_core_*<20 and 0.10<RMSd<0.20 nm, for the basin B; 14<Hb*_core_*<18 and 0.45<RMSd<0.60 nm, for the basin C.


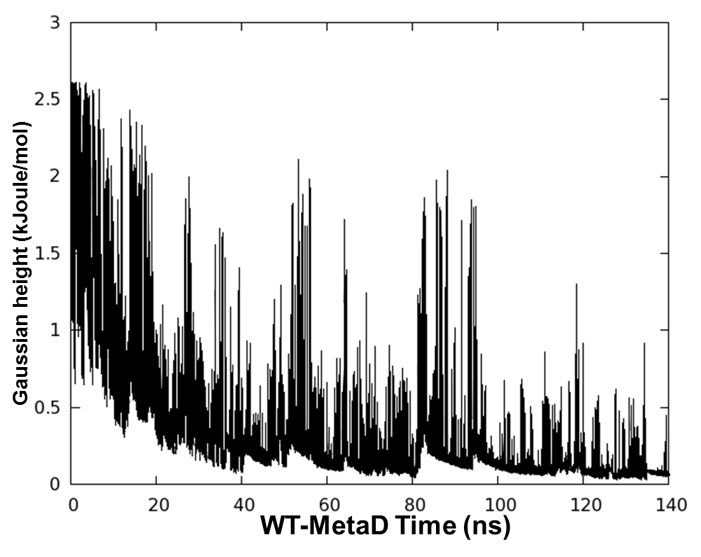


**Figure** **S8**. The evolution of the Gaussian height (W) added to the system along the WT-MetaD simulation as function of time.


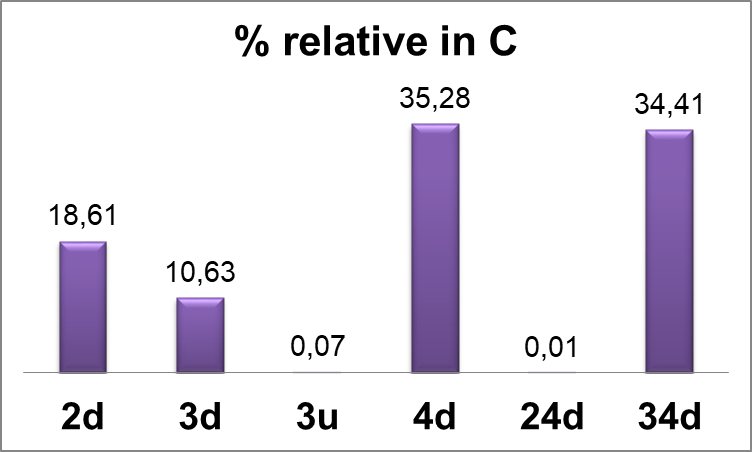


**Figure S9**. Percentage distribution of the slipped structure extracted from basin C of the reweighted FES.


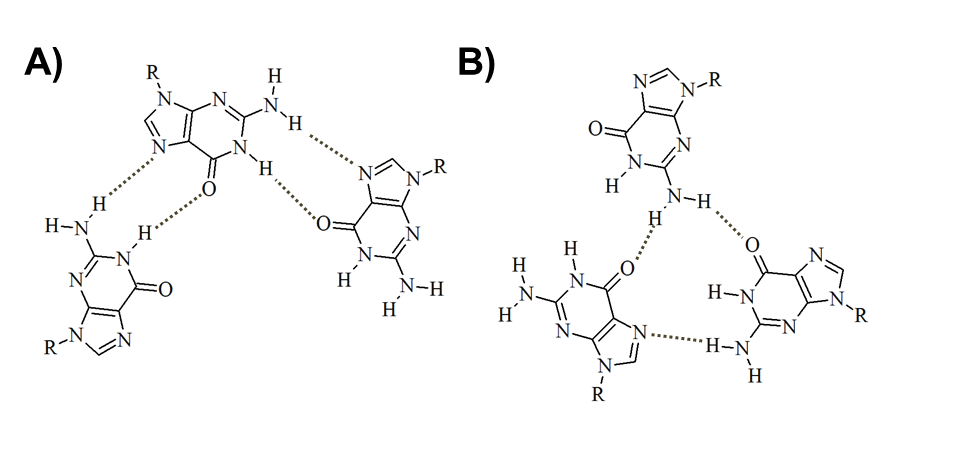


**Figure** **S10**. (**A-B**) 2D structure of two G-triads G4s found in the basin C. (**A**) An “*open*” G-triad is characterized by the conservation of the Hoogsteen-like hydrogen bond network and it was detected in **3d**, **3u** and **4d** conformations. (**B**) A “*closed*” G-triad showed a specific rearrangement of Hoogsteen-like hydrogen bonds and it was observed only in **2d** conformation.


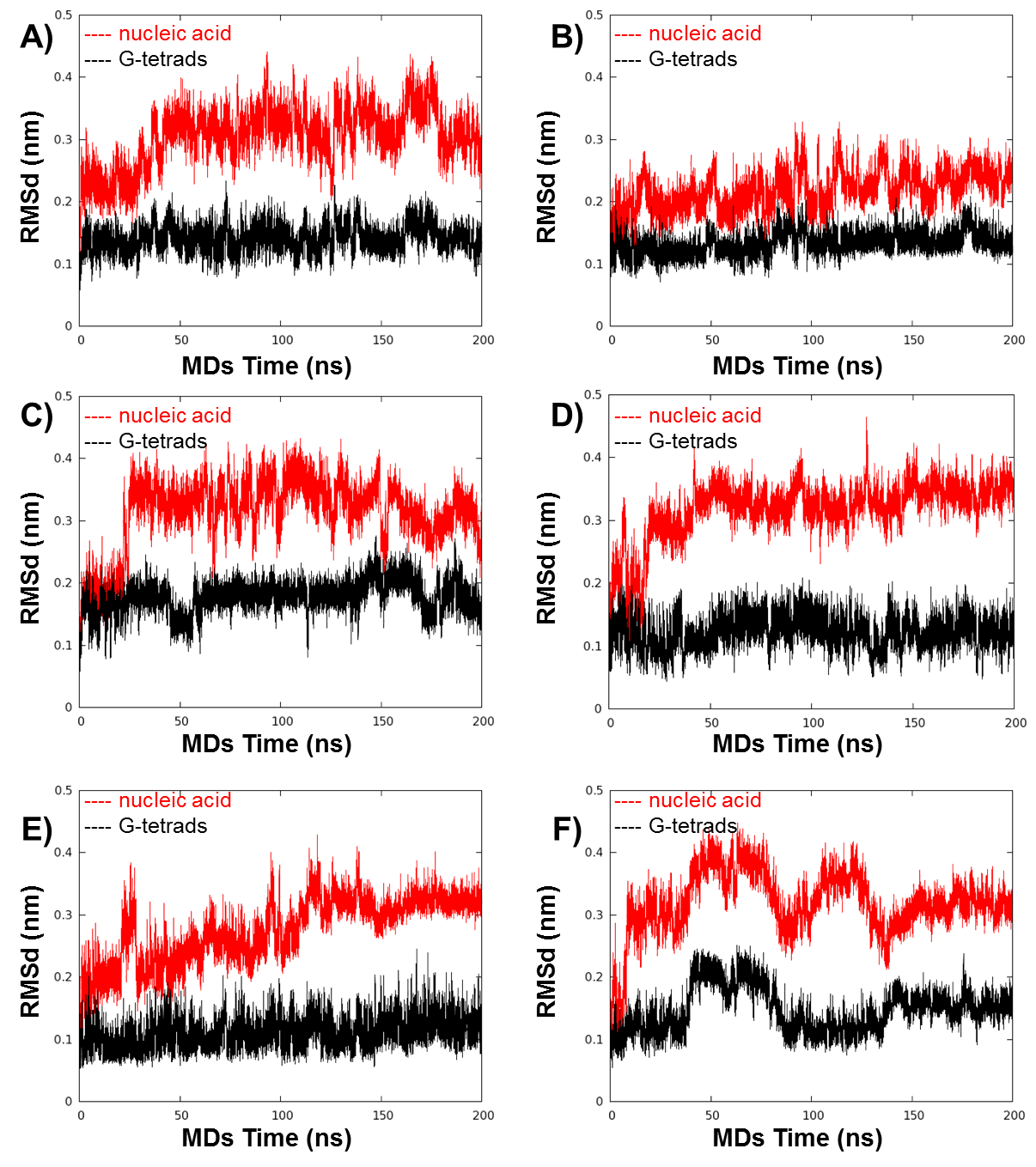


**Figure** **S11.** Plots showing the RMSd trends calculated during the MDs on the whole nucleic acid (red lines) and on the G-tetrads (black lines) of: **A)** **2d**, **B)** **3d**, **C) 3u**, **D) 4d**, **E) 24d** and **F) 34d** conformations of G4s composed of only two G-tetrads found in the basin C.


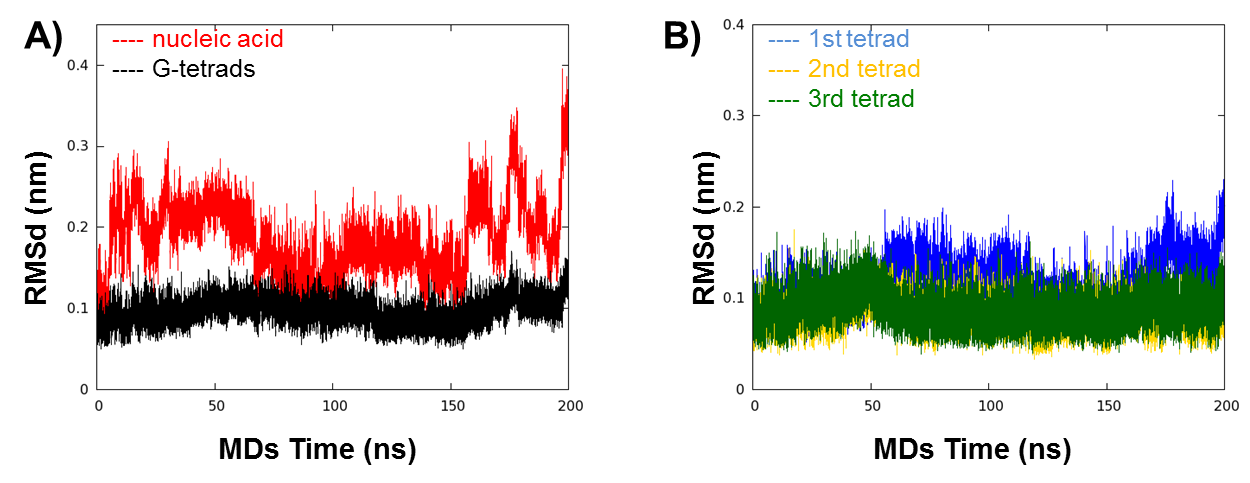
 **Figure** **S12.** **A)** Plot showing the RMSd trends calculated on the whole nucleic acid (red lines) and on the G-tetrads (black lines) of 1KF1_cry_ during the MDs. **B)** Plot showing the RMSd trends calculated on each tetrad of 1KF1_cry_ during the MDs.

**Geometrical instability of 24d**

The conformation **24d** is characterized by the simultaneous slippage of both the second and the fourth G-triplet toward the 3’-end direction. The main consequence of this movement is the absence of a G-triad both at 5’- and 3’-ends. In fact, while at 5’-end the guanine pair G2:G14 lies opposite to each other interacting by only two Hoogsteen Hydrogen bonds, at 3’-end G10 and G22 residues do not interact with each other. This kind of starting conformation (Figure S13A) and the absence of a well-defined G-triad seem to compromise the overall stability of the structure. In particular, after 100 ns of MDs, we observed a meaningful increase of the RMSd of the nucleic acid, especially in the last part of the simulation (see Figure S11D). In particular, the geometrical fluctuation of the guanine pair G2:G14 (Figure S13A), held together by two Hoogsteen Hydrogen bonds and interacting with A1 residue through stacking, is a reason behind the reduced stability of the overall **24d** structure. Moreover, at the 3’-end, G10 and G22 residues, after about 50 ns of MDs, changed their conformation, establishing an average of two Hoogsteen-like Hydrogen bonds that persist almost throughout the simulation. Therefore, the final **24d** conformation (Figure S13B) showed two guanine pairs placed outside the G-core at 3’ and 5’ ends forming two “caps”. In this conformation, only one K^+^ ion is able to maintain a stable bypiramidal coordination position among the eight oxygen of the guanines forming the two tetrads, throughout all the simulation.


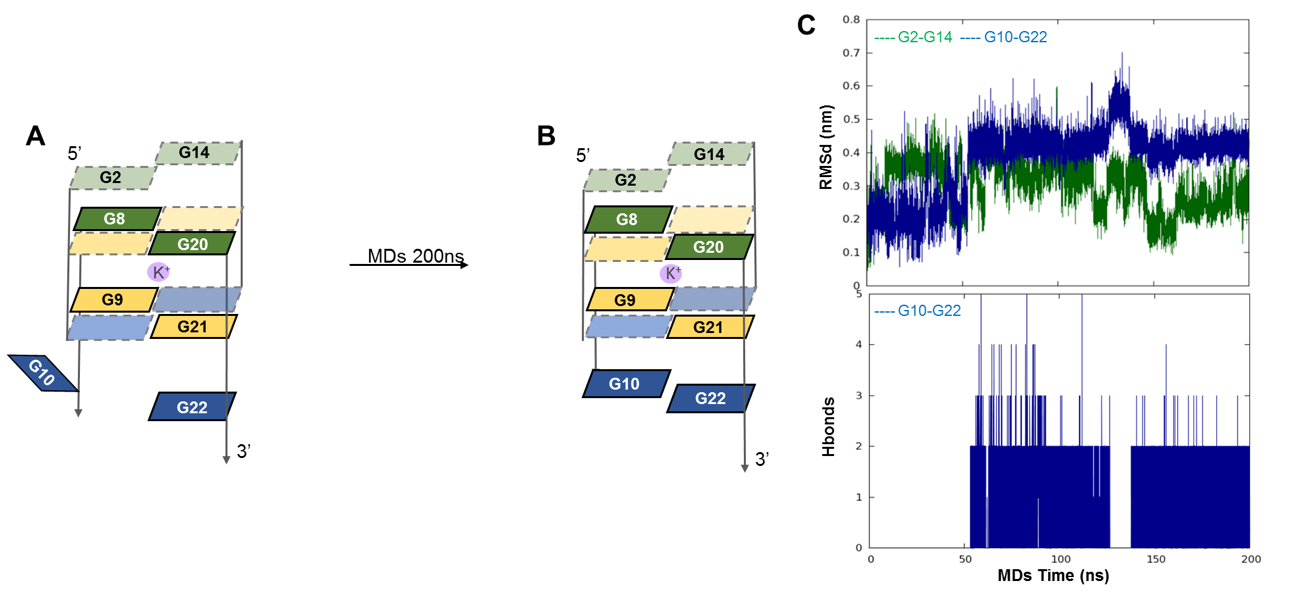


**Figure S13.** (**A**-**B**) Schematic representation of **24d** conformation before and after 200 ns of MDs, respectively. (**C**) (Upper panel) Plot showing the number of the Hydrogen bonds between the guanines G10:G22 (blue line) during the MDs. (Lower panel) Plot showing the RMSd trend calculated during the MDs on the heavy atoms of the couple of guanines G2:G14 (green line) and G10:G22 (blue line). It can be noted that after ~55 ns G10 moves towards G22 engaging an average of two hydrogen bonds. For clarity, the guanine residues not involved in the vertical slippage are shown in transparency with dashed grey lines.

**K^+^ ion affects the stability of the *“open”* G-triad in 3d conformation**

Unlike **3u** structure, **3d** arises from the vertical slippage of the third G-triplet toward the 3’-end direction, causing the formation of an *“open”* G-triad held by G2:G8:G20 residues. Globally, **3d** shows a good geometrical stability of the two/G-tetrads planes (see Figure S11B), while the G-triad at 5’-end seems more instable (Figure S14B, upper plot). Indeed, the RMSd trend of the G-triad shows a lot of fluctuations. In particular, it undergoes a slightly increase in the MDs interval between 20 and 60 ns and in the last 80 ns of the simulation (Figure S14B, upper plot). This behavior could be related to the coordination of the K^+^ ion that, in the first part of the simulation and in frames ranging from 60 to 110 ns, assumes a bipyramidal configuration by coordinating the seven O6 oxygen atoms of both the G-tetrad and the G-triad (Figure S14A, lower panel). By contrast, in the MDs interval between 20-60 ns, K^+^ is associated to a trigonal planar geometry, by coordinating only the three-guanine residues of the G-triad (Figure S14A, upper panel). Finally, we observed that K^+^ bypiramidal geometry stabilizes the G-triad, thanks also to the solvent contribution since one water molecule mimics the O6 oxygen atom of the missing G14 guanine by orienting its oxygen atom towards the center of the G-core channel and coordinating the K^+^. On the contrary, the K^+^ trigonal planar configuration does not favor the stability of the G-triad, thus inducing the loss of two Hoogsteen hydrogen bonds between G2:G8. Simultaneously to this event, in the site of the slipped guanine G14, we also observed an increase of the number of water bridges with G8:G20 through their two hydrogen atoms directed towards the channel of the G-triad.


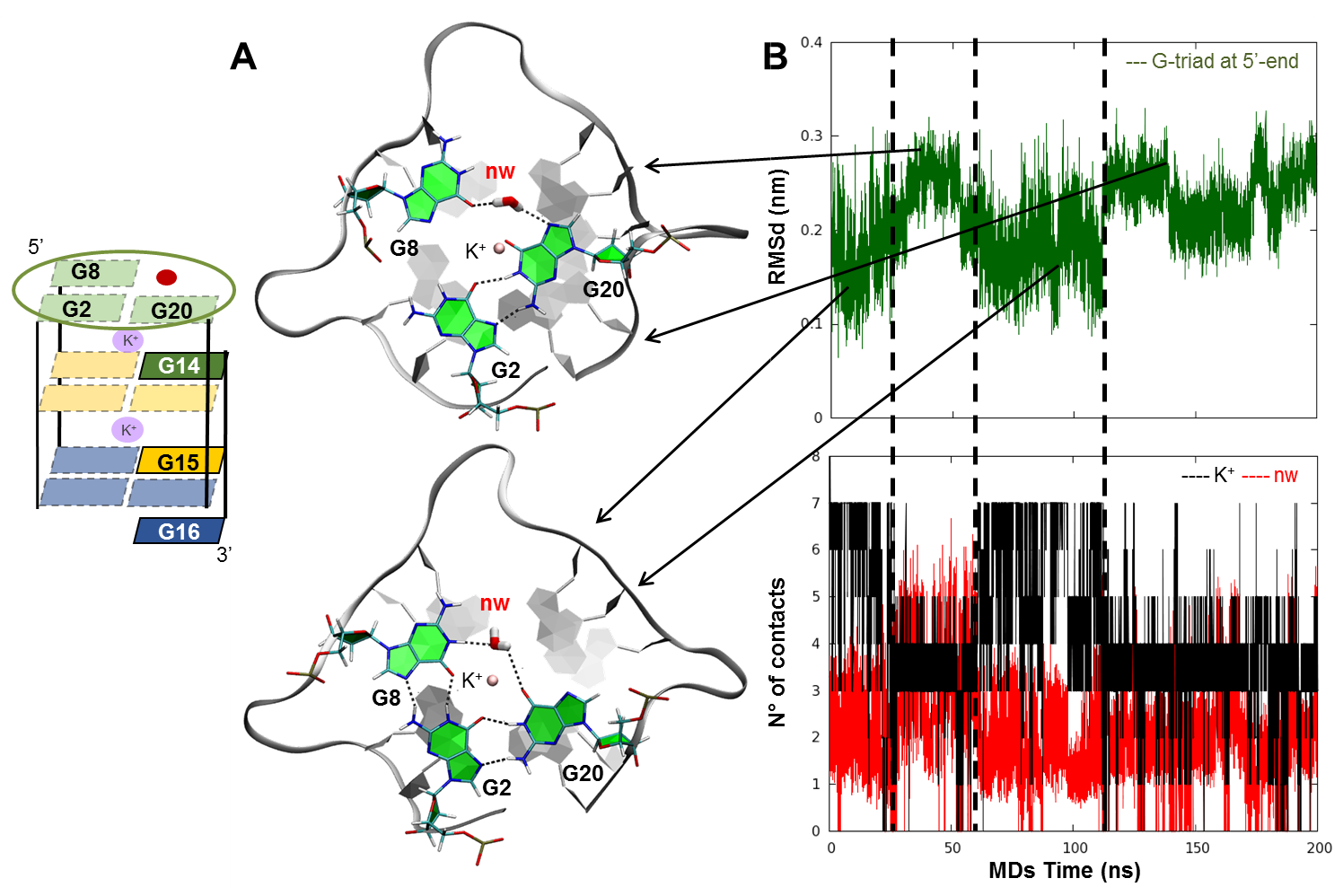


**Figure S14.** The stability of the *“open”* G-triad in **3d** structure affected by the coordination of the K^+^ ion. **(A)** 3D structures (upper panel) of the G-triad (G2:G8:G20) belonging to the conformation **3d,** characterized by two different orientations of the water molecule miming the missing guanine. In particular, the water can orient its hydrogen (upper panel) or (lower panel) oxygen atoms towards the channel of the G-core. (**B**) In the upper panel, plot showing the RMSd trend calculated during the MDs on the G-triad at 5’-end, formed by G2:G8:G20 residues (dark-green line). In the lower panel, plot showing the number of contacts between K^+^ ion and the guanines O6 oxygen atoms (black line) and the number of contacts between bulk water (nw) and the G8 and G20 residues (red line) during the MDs.

**3u conformation as single case of “open” G-triad at the 3’-end position.** Although the slippage movement toward the 5’-end direction is less favored, as demonstrated by its higher free energy value (Table 2), **3u** structure showed a stable G-tetrads, while loops rearranged in the first 20 ns of MDs before to stabilize the RMSd trend of the overall structure (see Figure S11C). In particular, the third loop has proven to be the portion of the structure with the largest fluctuations during the MDs, since its residues T17, T18 and A19 showed an average RMSF value of 0.36 nm (see Figure S15). It owns an “open” G-triad, consisting of residues G4:G10:G22 at the 3’-end position (Figure S16A, upper panel), and characterized by a good geometrical stability (Figure 16B, upper plot). In particular, we observed that its stability is linked by the presence of a K^+^ ion that coordinates O6 oxygen atoms of both the G-tetrad guanines G3:G9:G16:G21 and the G-triad residues G4:G10:G22 (Figure S16A, lower panel), as shown in the plot of Figure S16B (lower plot). Furthermore, our simulation revealed the ability of the G-triad to establish an average of three hydrogen bonds with a network of three water molecules (nw) mimicking the position of the slipped G16. In particular, the oxygen atom of the central water molecule coordinates the K^+^ ion bridging the G10:G22 and the other two adjacent water molecules, engaging through hydrogen bonds with N7 atom of G22 and the amino hydrogen of G10.

**
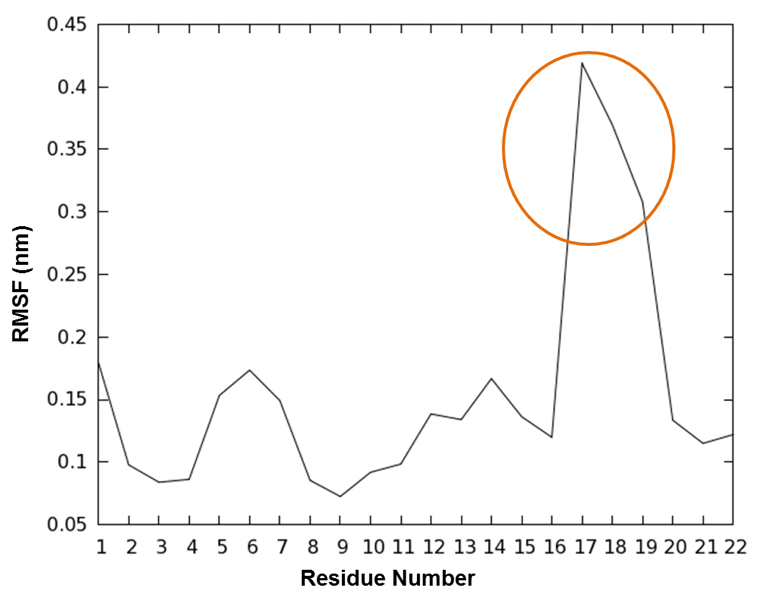
**

**Figure** **S15.** Plot showing the Root Mean Square Fluctuation (RMSF) calculated on all residues of **3u** structure during the MDs. The orange circle refers to the residues T17, T18 and A19 of the third loop with the largest fluctuations during the MDs.


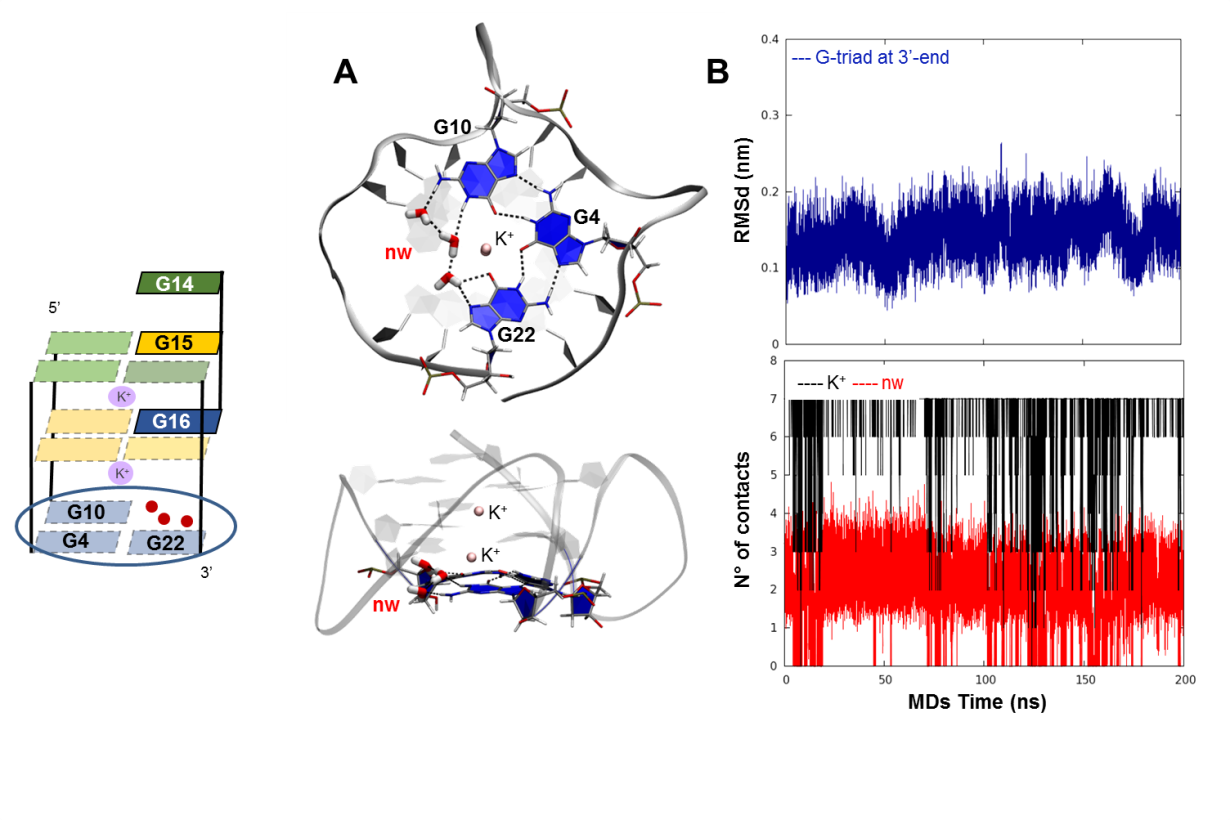


**Figure S16.** The only example for a stable “open” G-triad at the 3’-end position in **3u** structure. **(A)** Top (upper panel) and lateral (lower panel) views of the “open” G-triad formed at the 3’-end position by G4:G10:G22 residues found in **3u** conformation with the three water molecules network (nw) miming the G16 slipped residue. **(B)** In the upper panel, plot showing the RMSd trend calculated during the MDs on the G-triad at 3’-end, formed by G4:G10:G22 (blue line). In the lower panel, plot showing the number of contacts between K^+^ ion and the O6 oxygen atoms of the G-tetrad G3:G9:G9:G16:G21 and the G-triad G4:G10:G22 guanines (black line) and the number of contacts between bulk waters (nw) and the G10:G22 residues (red line) during the MDs.

**2d structure is the only characterized by a “closed” G-triad.** **2d** comes from the vertical slippage of the G-triplets formed by the G8:G9:G10 residues. Looking at the Figure S11A and analyzing the RMSd trend calculated on the heavy atoms of both the nucleic acid and the two G-tetrad planes, a good geometrical stability was reached after 40 ns of equilibration period during the MDs. **2d** conformation can be considered as a “transition” state from **24d** to 1KF1-like structure, since it derives from the vertical slippage of the second G-triplet of **24d** towards the 5’-end position. This movement allows G2 and G14 residues to reorganize themselves in order to form a typical G-triad in a “closed” conformation (see Figure S10B and Figure S17A, upper panel). This rearrangement seems linked to the absence of the coordinating K^+^ ion within the G-triad. Interestingly, in the early 10 ns of the MDs, we observed the inclusion of a water molecule (cw) into the channel between the G-triad and the below G-tetrad, with a decreased number of the hydrogen bonds of the G-triad (Figure S17A, lower panel). We realized that this event induces an increase of the RMSd value in the early 10 ns (Figure S17B, upper panel), due to a G-triad conformational change that allows a water molecule to enter into the G-core channel. Once this triad conformation is geometrically stable, the water molecule (cw) is capable of spreading in and out of the channel without causing major structural alterations, also thanks to the preserved hydrogen bonds between guanines of G-triad.


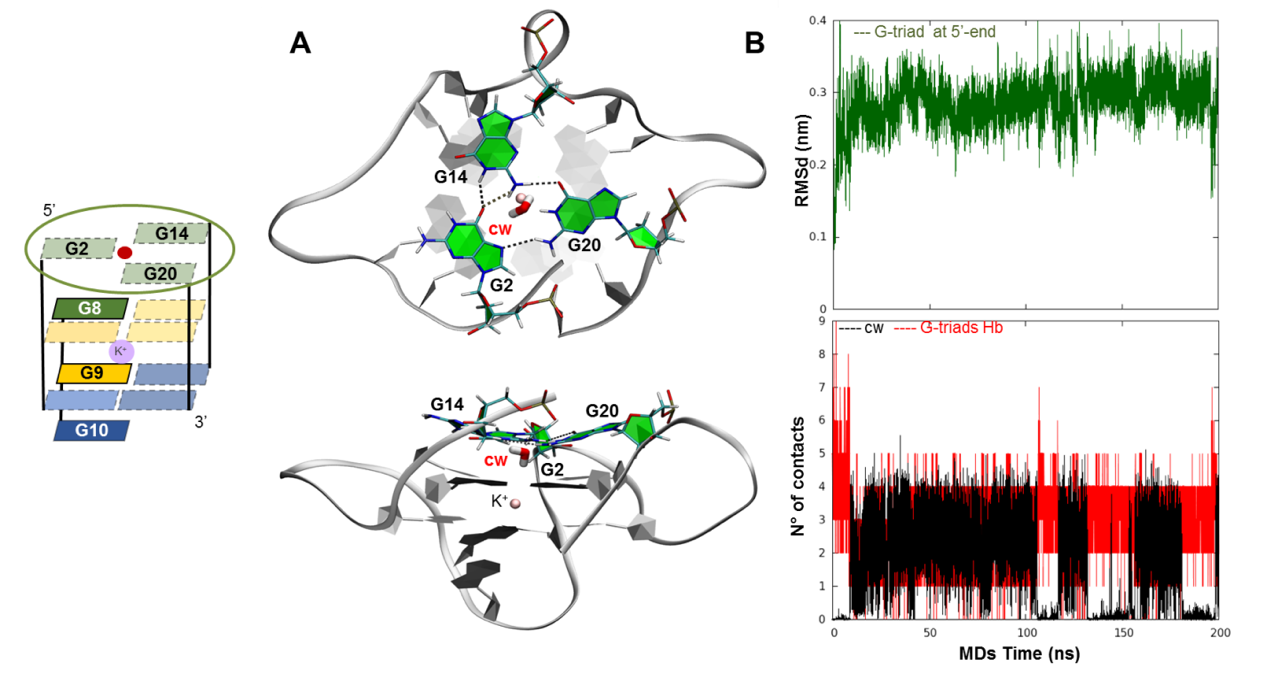


**Figure S17.** A coordinating water molecule in the central channel of **2d** structure. **(A)** Top (upper panel) and lateral (lower panel) views of the G-triad (G2:G14:G20) belonging to **2d** conformation, characterized by the presence of a water molecule in the central channel of the G-core, miming the K^+^ ion. **(B)** In the upper panel, plot showing the RMSd trend calculated during the MDs on the “closed” G-triad at 5’-end, formed by G2:G14:G20 (dark-green line). In the lower panel, plot showing the number of contacts between the water molecule, placed in the channel (cw), with the guanines O6 oxygen atoms (black line) and the number of hydrogen bonds established among the guanines of the G-triad (red line) during the MDs.

**The flexibility of the 3’-end in 34d structure and its geometrical instability. 34d** conformation is formed because of the simultaneous slippage of the third and the fourth triplets, leaving an adjacent pair of guanines in the 5’-end position (Figure S18A, upper panel) and another pair at the 3’-end (Figure S18A, lower panel), resulting in the absence of a well-defined G-triad. By analysing the RMSF plots, we observed a different geometrical stability between the 3’-end and the 5’-end (see Figure S19A). This different behaviour seems to be related to the high fluctuations of the third loop residues, especially T17, as shown in the RMSF analysis (see Figure S19A). The high flexibility of this loop affects also the stability of the 3’-end guanines G16:G22 (Figure S18B, upper plot) which, despite are involved in two hydrogen bonds (Figure S18B, middle plot), are the most unstable portion of the 3’-end due to the lack of a coordinating K^+^ ion (Figure S18B, lower plot). Unlike the loop residues of the 3’-end portion, the first and the second loop residues (A5:T6:T7 and A11:T12:T13, respectively) show less fluctuations (see Figure S19B) and are responsible for the stability of the 5’-end portion (see Figure S19A), especially of the guanine pair G2:G8 (Figure S18B, upper panel). The steady presence of a coordinating K^+^ ion between the two guanines and the below G-tetrad (Figure S18A, upper panel) contributes to further stabilize the 5’-end portion.


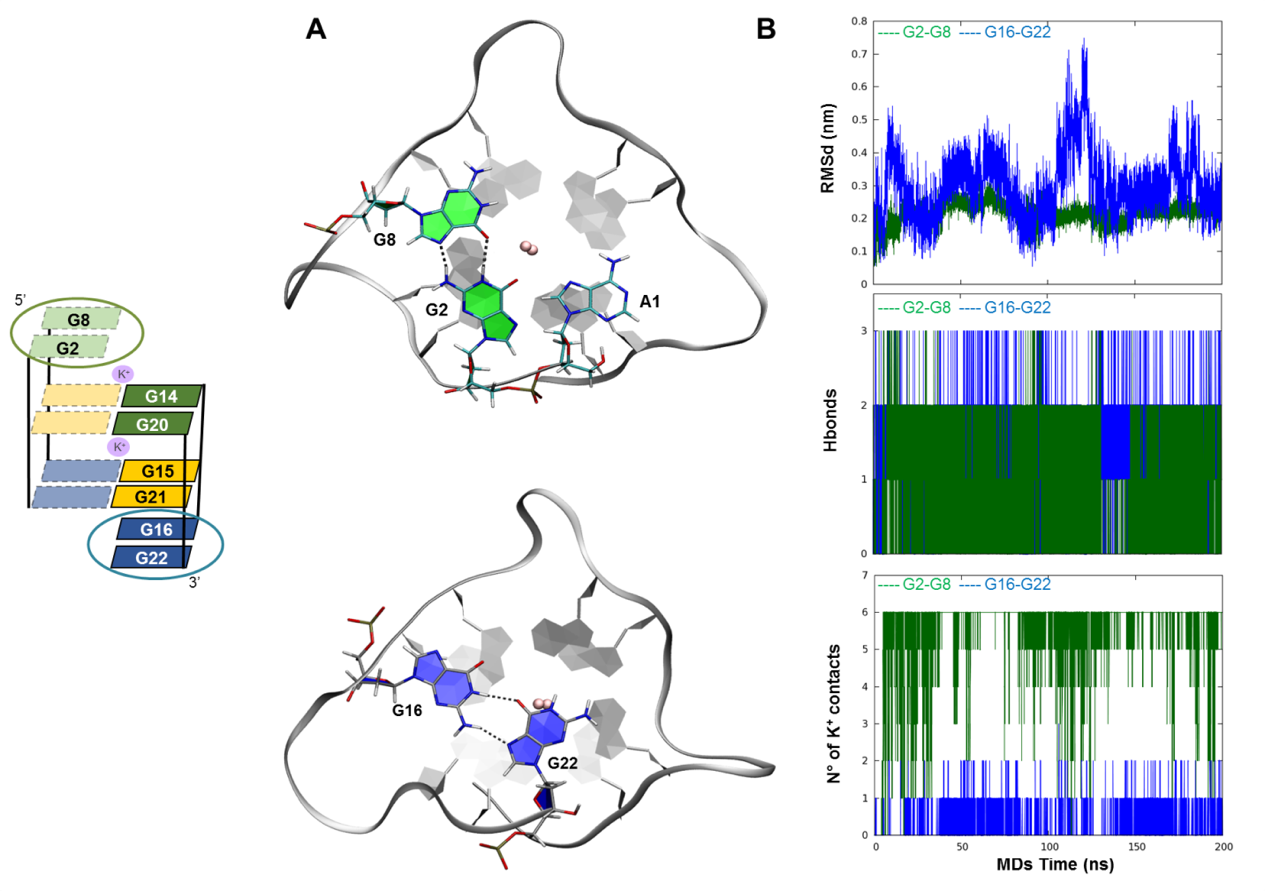


**Figure S18.** The different stability of G2:G8 and G16:G22 at 5’- and 3’-end position, respectively, in the 34d structure. (**A**) 3D structures of **34d** conformation in top (upper panel) and bottom (lower panel) perspective. (**B**) (Upper panel) Plot showing the RMSd trend calculated during the MDs on the residues G2:G8 (green line) and on the residues G16:G22 (blue line). (Central panel) Plot showing the number of Hbonds established between G2:G8 (green line) and between G16-G22 (blue line) residues. (Lower panel) Plot showing the number of contacts of K^+^ ion with the O6 oxygen atoms of G2:G8 (green line) and G16:G22 (blue line) residues during the MDs.


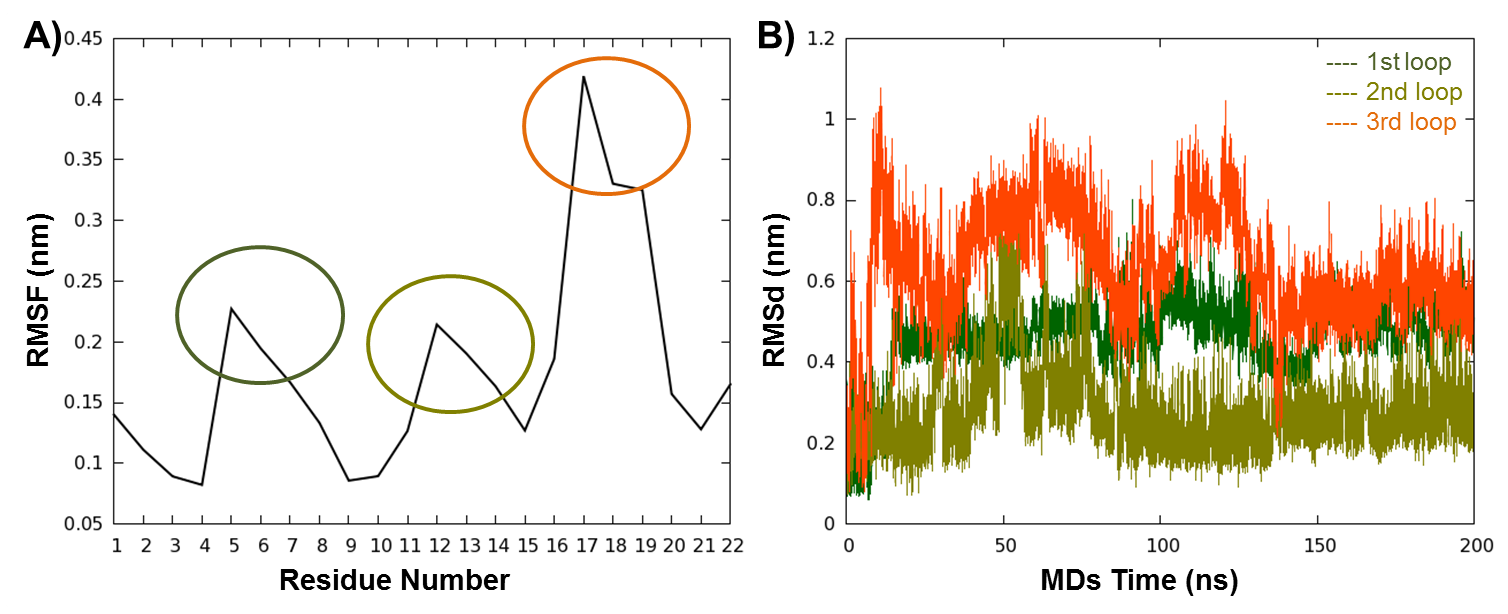


**Figure** **S19.** (**A**) Plot showing the Root Mean Square Fluctuation (RMSF) calculated on all residues of **34d** structure during the MDs. (**B**) Plot showing the RMSd trends calculated on the three loops of **34d** structure.

**
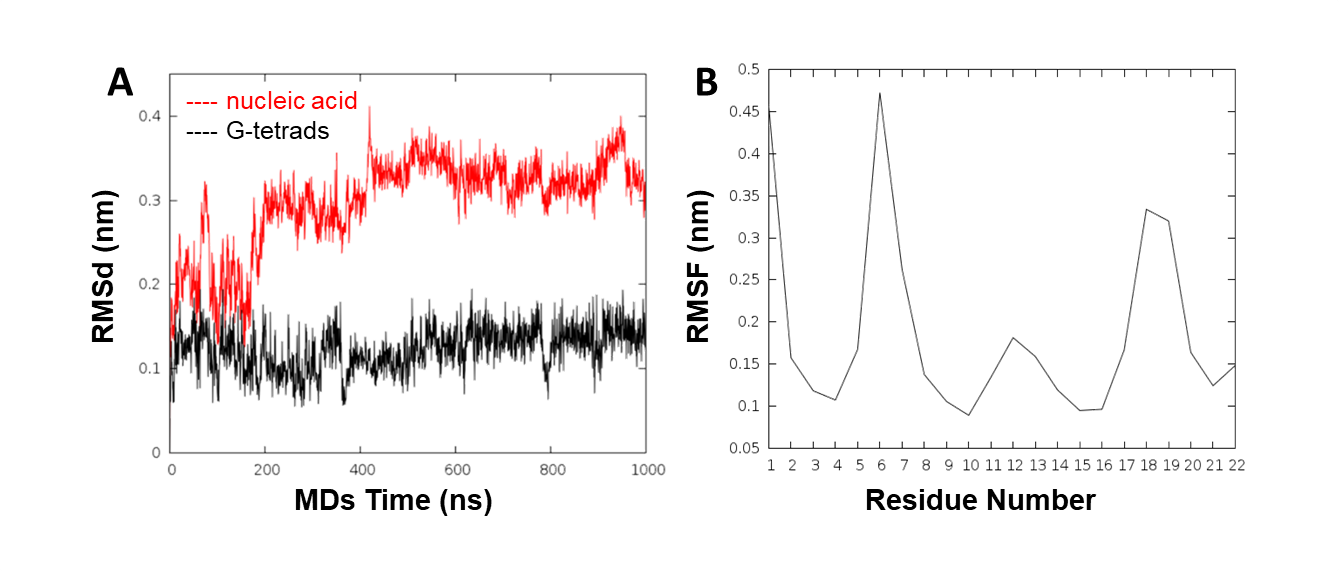
**

**Figure** **S20.** (**A**) Plots showing the RMSd trends calculated during the MDs on the whole nucleic acid (red lines) and on the G-tetrads (black lines) of **4d**. (**B**) Plot showing the Root Mean Square Fluctuation (RMSF) calculated on all residues of **4d** structure during the MDs.

**Potential restraint**

As potential restraint we used a wall for the value of the two CVs (Hb_core_ 1KF1 and π-π_core_), used during the WT-MetaD, which limits the region of the phase space accessible during the simulation. The restraining potential starts acting on the system when the value of the CV is lower (LWALL) than a certain limit LIMIT minus an off set OFF.

The functional form of this potential is the following:


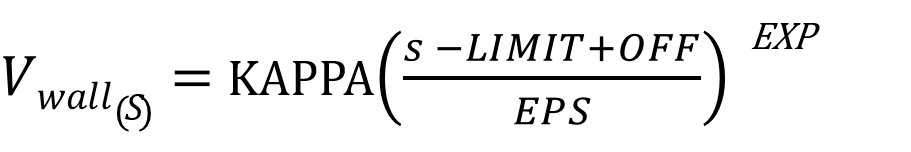


Where, KAPPA is an energy constant in internal unit of the code, EPS a rescaling factor and EXP the exponent determining the power law.

In particular, we used EXP = 2, EPS = 1:0 for both CVs, while the KAPPA was set to 50 and 100 for Hb_core_ 1KF1 and π-π_core_, respectively.

**1 2 3 4 5 6 7**


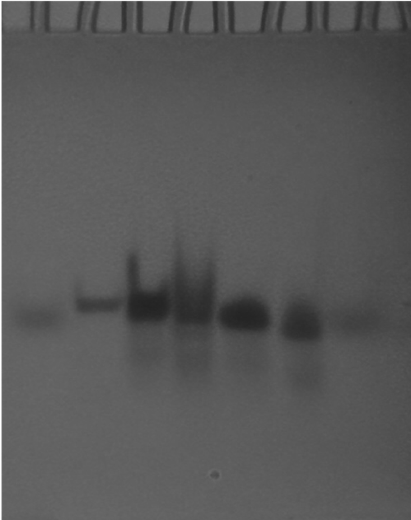


**Figure S21.** Native gel electrophoresis analysis in 20 mM potassium phosphate (pH 7.0) containing 70 mM KCl, 0.2 mM EDTA (K^+^ buffer). Lane **1**: bromophenol blue; Lane **2**: *h-tel_24_* in K^+^ buffer; Lane **3**: *h-tel_23_* in K^+^ buffer containing 40% acetonitrile (ACN); Lane **4**: *h-tel_22tr_* in K^+^ buffer containing 40% ACN; Lane **5**: *h-tel_23_* in K^+^ buffer containing 40% PEG200; Lane **6**: *h-tel_22tr_* in K^+^ buffer containing 40% PEG200; Lane **7**: bromophenol blue. Please note that lanes **5**, **6**, and **7** correspond respectively to lanes **1**, **2**, and **3** of the cropped Figure 4A of the main text.


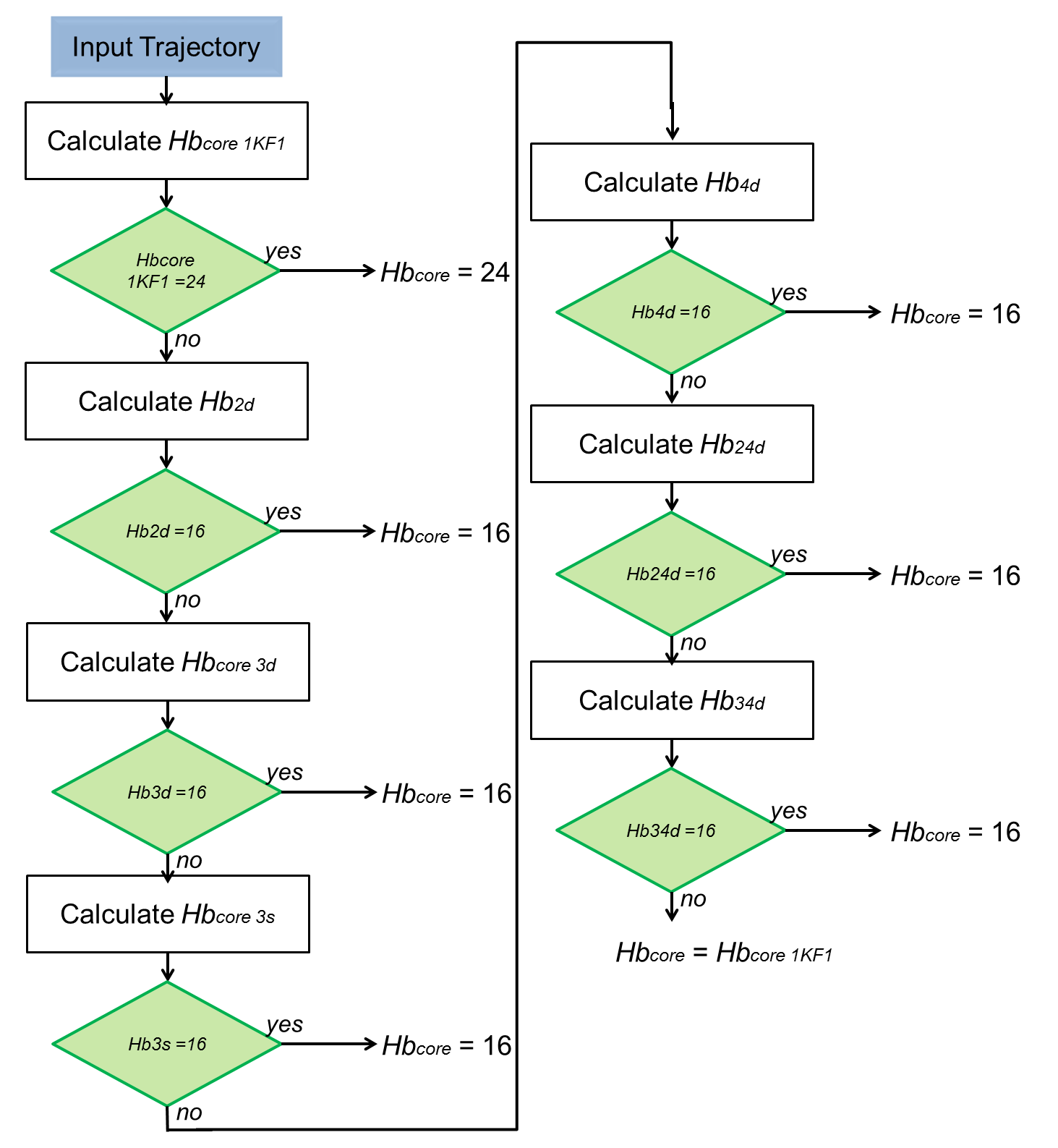


**Figure S22.** Flow diagram used for assigning the *Hb_core_* value to each trajectory structure.

**Supplementary movie SM1**

The vertical slippage mechanism of the fourth G-triplet formed from residues G20:G21:G22 (structure **4d**) observed during 140 ns of WT-MetaD.

After 10 seconds of movie, it can be observed the vertical slippage of G20:G21:G22 (coloured as green, yellow and green stick, respectively) toward the 3’-end and the formation of a stable “open” G-triad G8:G14:G2 (coloured as green wireframes).

**Supplementary movie SM2**

1µs of MDs of the structure **4d** showing the *syn-anti* conversion of the A1 nucleobase and its positioning on the same plane of the G-triad. This event is crucial for the whole stability of **4d.**

**References**

1. Giberti, F., Salvalaglio, M., Mazzotti, M. and Parrinello, M. Insight into the nucleation of urea crystals from the melt. *Chem. Eng. Sci.*, **121**, 51-59 (2015).

2. Islam, B., Sgobba, M., Laughton, C., Orozco, M., Sponer, J., Neidle, S. and Haider, S. Conformational dynamics of the human propeller telomeric DNA quadruplex on a microsecond time scale. *Nucleic Acids Res*., **41**, 2723-2735 (2013).

3. Barducci, A., Bussi, G. & Parrinello M. Well-tempered metadynamics: a smoothly converging and tunable free-energy method. *Phys Rev Lett.* **100**, 020603 (2008).
